# *HOTAIR* and its surrogate DNA methylation signature indicate carboplatin resistance in ovarian cancer

**DOI:** 10.1186/s13073-015-0233-4

**Published:** 2015-10-24

**Authors:** Andrew E. Teschendorff, Shih-Han Lee, Allison Jones, Heidi Fiegl, Marie Kalwa, Wolfgang Wagner, Kantaraja Chindera, Iona Evans, Louis Dubeau, Arturo Orjalo, Hugo M. Horlings, Lukas Niederreiter, Arthur Kaser, Winnie Yang, Ellen L. Goode, Brooke L. Fridley, Richard G. Jenner, Els M.J.J. Berns, Elisabeth Wik, Helga B. Salvesen, G. Bea A. Wisman, Ate G.J. van der Zee, Ben Davidson, Claes G. Trope, Sandrina Lambrechts, Ignace Vergote, Hilary Calvert, Ian J. Jacobs, Martin Widschwendter

**Affiliations:** Statistical Genomics Group, UCL Cancer Institute, University College London, London, UK; CAS Key Lab of Computational Biology, CAS-MPG Partner Institute for Computational Biology, Shanghai Institute for Biological Sciences, Chinese Academy of Sciences, Shanghai, China; Department of Women’s Cancer, UCL Elizabeth Garrett Anderson Institute for Women’s Health, University College London, London, UK; Sloan Kettering Institute, Cancer Biology & Genetics Program, New York, NY USA; Department of Gynaecology and Obstetrics, Innsbruck Medical University, Innsbruck, Austria; Helmholtz-Institute for Biomedical Technology, Stem Cell Biology and Cellular Engineering, RWTH Aachen University Medical School, Aachen, Germany; Department of Pathology, USC/Norris Comprehensive Cancer Center, Keck School of Medicine of University of Southern California, Los Angeles, CA USA; Biosearch Technologies, Novato, CA USA; Department of Pathology and Laboratory Medicine, University of British Columbia, Vancouver, BC Canada; Department of Medicine, Addenbrooke’s Hospital, University of Cambridge, Cambridge, UK; Department of Molecular Oncology, British Columbia Cancer Agency Research Centre, Vancouver, Canada; Department of Health Sciences Research, Mayo Clinic College of Medicine, Rochester, MN USA; Biostatistics and Informatics Shared Resource, The University of Kansas Cancer Center, University of Kansas Medical Center, Kansas City, KS USA; UCL Division of Infection and Immunity, University College London, London, UK; Department of Medical Oncology, Erasmus MC-Cancer Center, Rotterdam, The Netherlands; Centre for Cancer Biomarkers, CCBIO, Department of Clinical Science, University of Bergen, Bergen, Norway; Department of Pathology, Haukeland University Hospital, Bergen, Norway; Department of Obstetrics and Gynaecology, Haukeland University Hospital, Bergen, Norway; Department of Gynaecological Oncology, University of Groningen, University Medical Center Groningen, Groningen, Netherlands; Division of Pathology, Oslo University Hospital, Norwegian Radium Hospital, Oslo, Norway; Department of Gynaecological Oncology, Oslo University Hospital, Norwegian Radium Hospital, Oslo, Norway; Division of Gynecologic Oncology, Department of Obstetrics and Gynecology and Leuven Cancer Institute, University Hospitals Leuven, Katholieke Universiteit Leuven, Leuven, Belgium; Drug Development Group, UCL Cancer Institute, University College London, London, UK; University of Manchester, Manchester, UK; University of New South Wales, Sydney, Australia

## Abstract

**Background:**

Understanding carboplatin resistance in ovarian cancer is critical for the improvement of patients’ lives. Multipotent mesenchymal stem cells or an aggravated epithelial to mesenchymal transition phenotype of a cancer are integrally involved in pathways conferring chemo-resistance. Long non-coding RNA *HOTAIR* (HOX transcript antisense intergenic RNA) is involved in mesenchymal stem cell fate and cancer biology.

**Methods:**

We analyzed *HOTAIR* expression and associated surrogate DNA methylation (DNAme) in 134 primary ovarian cancer cases (63 received carboplatin, 55 received cisplatin and 16 no chemotherapy). We validated our findings by *HOTAIR* expression and DNAme analysis in a multicentre setting of five additional sets, encompassing 946 ovarian cancers. Chemo-sensitivity has been assessed in cell culture experiments.

**Results:**

*HOTAIR* expression was significantly associated with poor survival in carboplatin-treated patients with adjusted hazard ratios for death of 3.64 (95 % confidence interval [CI] 1.78–7.42; *P* < 0.001) in the discovery and 1.63 (95 % CI 1.04–2.56; *P* = 0.032) in the validation set. This effect was not seen in patients who did not receive carboplatin (0.97 [95 % CI 0.52–1.80; *P* = 0.932]). *HOTAIR* expression or its surrogate DNAme signature predicted poor outcome in all additional sets of carboplatin-treated ovarian cancer patients while *HOTAIR* expressors responded preferentially to cisplatin (multivariate interaction *P* = 0.008).

**Conclusions:**

Non-coding RNA *HOTAIR* or its more stable DNAme surrogate may indicate the presence of a subset of cells which confer resistance to carboplatin and can serve as (1) a marker to personalise treatment and (2) a novel target to overcome carboplatin resistance.

**Electronic supplementary material:**

The online version of this article (doi:10.1186/s13073-015-0233-4) contains supplementary material, which is available to authorized users.

## Background

Late stage presentation aside, carboplatin resistance in ovarian cancer is the key obstacle to improving survival in this disease [1]. The observation that re-treatment with platinum-based drugs 6–12 months after primary response proved to be successful in a certain percentage of patients [2] is consistent with the idea that platinum sensitivity can be modulated by both cancer cell-autonomous and non-autonomous factors. For both these factors stromal/mesenchymal differentiation is crucially important. Epithelial–mesenchymal transition (EMT) in ovarian cancer cells is associated with platinum resistance [3–5]. On the other hand the cancer cell-autonomous tumour stroma and mesenchymal stem cells (MSCs) — mainly recruited from the bone marrow [6] — might play an important role in ovarian cancer biology [7, 8]. Recently, bone marrow-derived MSCs and embryonic fibroblasts, but not more extensively differentiated stromal cells, have been shown to induce platinum resistance in ovarian cancer [9].

Long non-coding RNAs are known to epigenetically remodel chromatin states and influence gene transcription in normal and cancer tissue towards stromal/mesenchymal differentiation [10–13]. Aberrant expression of non-coding RNAs has been observed in numerous diseases, including cancer [14], yet their precise contribution to disease aetiology and biology is far from clear. *HOX* antisense transcript intergenic RNA (*HOTAIR*), transcribed from the *HOXC* locus, represses transcription by recruiting polycomb repressive complex 2 (PRC2) to specific polycomb group target (PCGT) genes, in particular to those normally targeted by PRC2 in embryonic fibroblasts [10].

In stem cells, PCGTs are repressed through PRC2 occupancy and PCGTs important for specialised cell identities become de-repressed upon differentiation [15, 16]. We and others have shown that the promoters of these stem cell PCGTs become methylated and silenced in cancer [17–20]. It was recently reported that the expression of *HOTAIR* is increased in various cancer entities and that high levels of expression correlate with cancer invasiveness, metastases and poor prognosis [10, 21]. A recent systematic review of 19 papers (including a total of 2255 patients) demonstrates consistently that *HOTAIR* expression is a poor prognostic marker across a large set of cancers [22]. It is unclear, however, whether *HOTAIR* is associated with an aberrant DNA methylation profile in cancer and whether this robust DNA-based imprint mediates resistance to specific drugs.

Here we tested the hypothesis that *HOTAIR* RNA expression, or a *HOTAIR*-associated DNA methylation (DNAme) signature, as surrogates for mesenchymal differentiation, serve as markers for carboplatin resistance in primary ovarian cancer.

## Methods

### Ovarian cancer data sets

We analyzed six different data sets, details of which are provided in Additional file 1.

The first data set consisted of primary ovarian cancer samples (*n* = 134, 24–87 years, median 62.7 years at diagnosis; Additional file 2) treated at the Innsbruck Medical University, denoted “INNSBRUCK”. Clinicopathological features are shown in Table 1. The study was approved by the ethical committee of the Medical University Innsbruck (reference number UN4044). For the majority of patients exemption from obtaining informed consent was received as the majority of ovarian cancer patients were dead at the time the application was evaluated. The median survival time was 3.8 years. *HOTAIR* expression was measured in all 134 samples. DNA methylation data are available as Additional file 3.

**Table 1:** Clinicopathological features of patients from the INNSBRUCK data set stratified according to *HOTAIR* expression

Characteristics		*HOTAIR* RNA expression		
		Negative	Positive	*P* value^a^
		(*n* = 62)	(*n* = 72)	
Age				0.166
≤ 62.7 years (median age)	67	35	32	
> 62.7 years (median age)	67	27	40	
FIGO stage				0.733
I/II	37	18	19	
III/IV	97	44	53	
Tumour grade				0.182
I/II	78	33	45	
III	51	28	23	
Unknown	5	1	4	
Histology				0.145
Serous cancer	56	28	28	
Mucinous cancer	43	19	24	
Endometrioid cancer	24	14	10	
Clear cell cancer	6	1	5	
Not classifiable ovarian tissue	2	0	2	
Fallopian tube cancer	3	0	3	
Residual disease after surgery				0.308
No residual disease	46	22	24	
Residual disease ≤ 2 cm	36	19	17	
Residual disease > 2 cm	45	20	25	
Unknown	7	1	6	
Chemotherapy				0.453
Not performed	16	6	10	
Performed	118	56	62	
Health status				0.074
No relapse	56	31	25	
Relapse	78	31	47	
Survival status				0.023
Alive	39	24	15	
Dead	95	38	57	

^a^ *P* values were calculated with the use of the Chi square test

The second data set consisted of primary ovarian cancer samples (*n* = 175, 21–83 years, median 60.0 years at diagnosis; Additional file 4) treated at the University Medical Center in Groningen [23], denoted “GRONINGEN”. The median survival time in this set, which consisted only of stage III/IV patients, was 2.1 years. For these 175 samples, we measured *HOTAIR* expression. Of the 175 samples, 157 received carboplatin only, whilst 18 received cisplatin instead. For 114 of these samples there were matched mRNA array expression profiles available (Operon Human v.3 ~ 35 K 70-mer two-color oligonucleotide arrays, Gene Expression Omnibus accession [GEO:GSE13876]). Patients gave informed consent for collection and storage of tissue samples in a tissue bank for future research. All relevant patient data were retrieved and transferred into an anonymous, password-protected database. The patients’ identity was protected by study-specific, unique patient codes and their true identity was only known to two dedicated data managers. According to Dutch regulations, these precautions meant no further institutional review board approval was needed.

The third data set consisted of primary ovarian cancer samples (*n* = 342, serous ovarian cancers, median 58 years at diagnosis; 316 received carboplatin-based chemotherapy with the rest received cisplatin or were untreated), analyzed within The Cancer Genome Atlas (TCGA) program, and for which Illumina Infinium 27 k DNAme data were publicly available [24] (Additional file 5), denoted “TCGA”. The median survival time was 2.6 years.

The fourth data set consisted of primary ovarian cancer samples from three European Cancer centres (Leuven, Oslo, Rotterdam; *n* = 206, median 58 years at diagnosis; Additional file 6), denoted “EUROPE”. The median survival time was 3.4 years. For this data set, Illumina Infinium 450 k DNAme data were available for 121 carboplatin- and 85 cisplatin-treated patients. Data are deposited in the GEO, accession [GEO:GSE72021]. The study from Rotterdam has been approved by the local medical ethics committee (MEC-2008-183), performed in accordance with the Code of Conduct of the Federation of Medical Scientific Societies in the Netherlands. The Regional Committee for Medical Research Ethics in Norway approved the study (for patients diagnosed before 2007, exemption from obtaining informed consent was received as the majority of ovarian cancer patients were dead at the time the application was evaluated; patients diagnosed after 2007 signed general consent allowing for use of the tumours for research purposes). Written informed consent for the use of tumour tissue and prospective clinical data collection was obtained from all patients and approved by the Leuven ethics committee.

The fifth data set consisted of primary ovarian cancer samples from Bergen (*n* = 49) with 40 receiving carboplatin and 9 untreated (no chemotherapy), denoted “BERGEN”. Patients were included in the study after written informed consent, approved by the Regional Research Ethics Committee in Medicine. For samples from this cohort, we measured *HOTAIR* expression.

The sixth data set consisted of primary ovarian cancer samples from Rochester-Mayo (*n* = 174), denoted “ROCHESTER-MAYO”. All 174 patients received carboplatin and for these samples we measured DNAme using Illumina 450 k beadarrays. All patients gave informed consent and the Mayo Clinic Institutional Review Board approved the study. The data are available from Dr Ellen Goode at the Department of Health Sciences Research, Mayo Clinic, Rochester, USA, upon request.

Our research conformed to the Helsinki Declaration.

### *HOTAIR* expression

Total RNA was extracted by the acid guanidium thiocyanate-phenol-chloroform method [25]. Reverse transcription of RNA was performed as previously described [26]. Primers and probes for *HOTAIR* were designed using Primer Express (Applied Biosystems, Foster City, CA, USA). Samples in which *HOTAIR* was not amplified by real-time PCR after 45 cycles were classified as negative (*HOTAIR*-ve; Additional file 1).

### DNA methylation analysis

DNA was isolated from tissue samples using the Qiagen DNeasy Blood and Tissue Kit (Qiagen Ltd, UK, 69506) and 600 ng was bisulphite converted using the Zymo Methylation Kit (Zymo Research Inc, USA, D5004/8). Genome-wide methylation analysis was performed using the Illumina Infinium Methylation 27 K or 450 K beadchip (Illumina Inc., USA, WG-311-1201 and WG-314-1003). Analysis and quality control were performed as previously described [19, 27, 28].

### *HOTAIR* overexpression in ovarian cancer cell lines

The SKOV3IP cells were stably transduced with *HOTAIR* and LacZ constructs, kindly provided by Dr Chang (Stanford) [10] and single clones of *HOTAIR*/LacZ overexpressing cells were used for experiments (Additional file 1). Cells were treated with cisplatin (0.5–18 μM) or carboplatin (10–160 μM) for 3 days and analysed by the cell survival MTT assay (Sigma).

### Statistics

To test for differences in categorical variables, we used the Chi square test. Impact of *HOTAIR* expression on ovarian cancer survival was ascertained using log rank test and Kaplan Meier curves. To demonstrate the effect of *HOTAIR* expression on DNAme patterns, we first performed univariate analysis to rank CpGs according to their association with *HOTAIR* expression (Additional file 1). We used 10-fold internal cross-validations to identify an optimal *HOTAIR*-associated DNAme signature consisting of 67 CpGs (Additional file 7) at an estimated false discovery rate (FDR) of approximately 0.17. To build a single-sample classifier from this signature, a cutoff was optimized using receiver operating characteristic (ROC) analysis. This same cutoff was then used to assign samples from independent cohorts into two groups exhibiting high and low correlations with the *HOTAIR* DNAme signature. For the PCGT enrichment analysis we relaxed the threshold of the DNAme signature to include the top 500 ranked CpGs (FDR < 0.3), and divided the 500 into the 233 which were hypermethylated and the 267 which were hypomethylated in high *HOTAIR* expressors.

## Results

### *HOTAIR* expression in primary ovarian cancer is not associated with clinicopathological features

We analyzed *HOTAIR* expression in 134 primary ovarian cancer samples (INNSBRUCK) and found that 72 were positive and 62 were negative for *HOTAIR*. Consistent with our findings that only a subset of cells may express *HOTAIR* in ovarian cancer tissue, the cycle threshold (ct) values in the positive samples were very low (mean ct 37.4) compared with the *TBP* reference gene (mean ct 27.4). On assessing the clinicopathological characteristics of the sample set, survival status was the only characteristic that was significantly associated with *HOTAIR* expression, i.e., those patients whose tumours were *HOTAIR* + ve had a poor outcome compared with *HOTAIR*-ve tumours (*P* = 0.023, Table 1).

### Association between *HOTAIR* expression and poor outcome is restricted to carboplatin-treated patients

**Fig. 1:**
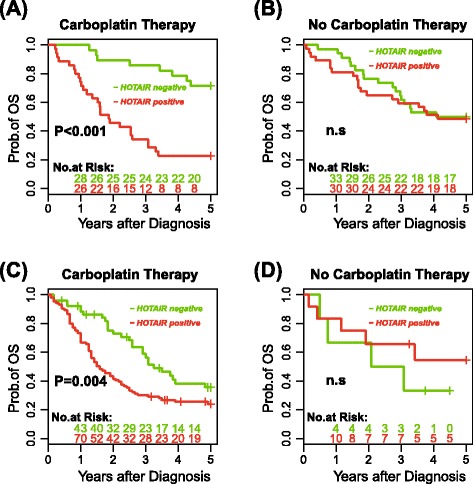
Kaplan-Meier survival estimates in patients from the INNSBRUCK set (**a**, **b**) and from the GRONINGEN set (**c**, **d**) who received carboplatin-based chemotherapy (**a**, **c**) and in patients who received no carboplatin-based chemotherapy (**b**, **d**) according to the presence or the absence of *HOTAIR* RNA in their ovarian cancer tumour specimens. The survival analysis was performed in the INNSBRUCK set based on the patients who did receive carboplatin-based chemotherapy (*n* = 63) referred to as “*Carboplatin Therapy*” and the 71 ovarian cancer patients who received cisplatin-based (*n* = 55) or no chemotherapy (*n* = 16) referred to as “*No Carboplatin Therapy*”. In the GRONINGEN set the survival analysis was performed based on the ovarian cancer patients who did receive carboplatin-based chemotherapy (*n* = 157), referred to as “*Carboplatin Therapy*” and patients who received cisplatin-based chemotherapy (*n* = 18), referred to as “*No Carboplatin Therapy*”. *n.s* not significant, *OS* overall survival

In order to test the hypothesis that *HOTAIR* is linked to carboplatin resistance we analyzed survival in patients who received carboplatin both alone or as part of a combination therapy (‘carboplatin’ group) compared with those who received cisplatin or no chemotherapy (‘no carboplatin group’) in the INNSBRUCK set. We note that there was no difference in any clinicopathological feature or survival between the two groups (Additional file 2). *HOTAIR* expression was significantly associated with both risk of relapse (hazard ratio (HR) 4.46 [*P* < 0.001] and 3.38 [*P* = 0.003] in uni- and multivariate analysis, respectively) and of death (HR 4.02 [*P* < 0.001] and 3.64 [*P* < 0.001] in uni- and multivariate analysis, respectively) in the carboplatin group (Fig. 1a; Additional file 8), whereas *HOTAIR* expression was not associated with survival in the ‘no carboplatin’ group (Fig. 1b; Additional file 8).

Only 34 % of patients who received carboplatin and whose tumours expressed *HOTAIR* survived the first 3 years post-diagnosis, whereas 85 % of those patients who received carboplatin, but whose tumours were *HOTAIR*-ve, survived during the same time period (Fig. 1a). Nearly half of the carboplatin-treated patients also received paclitaxel, and in both groups, i.e., single agent carboplatin and combined carboplatin/paclitaxel, *HOTAIR* was significantly associated with poor outcome (log rank *P* value = 0.006 and 0.003, respectively), again indicating the interaction of *HOTAIR* with carboplatin but not with cisplatin or paclitaxel.

To validate these findings we analyzed 175 ovarian cancer samples from Groningen [23] and 49 samples from Bergen and again confirmed that *HOTAIR* expression is a poor prognostic factor specifically in carboplatin-treated patients (Fig. 1c, d; Additional files 9 and 10).

### *HOTAIR* expression is associated with a DNAme profile enriched for PCGTs and associated with multipotent MSCs

**Fig. 2:**
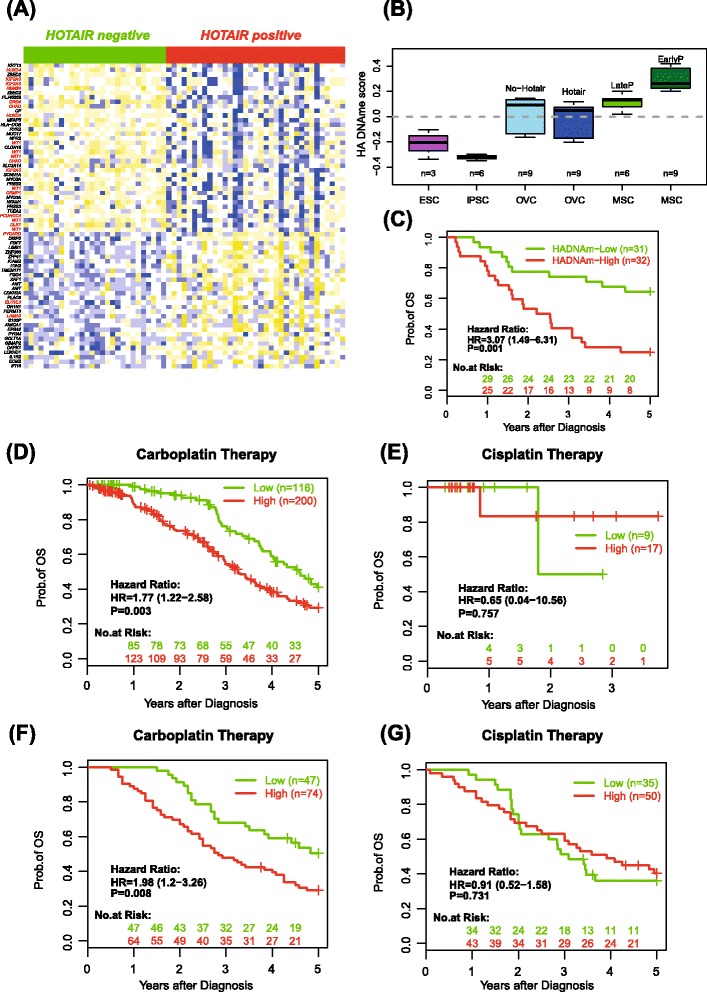
*HOTAIR*-associated DNA methylation signature predicts survival in carboplatin-treated ovarian cancer patients. **a** Heatmap of the 67 CpG DNAme signature (gene symbol for PCGTs in *red*) strongly associated with *HOTAIR* expression in ovarian cancers. CpG methylation profiles were centred to mean zero and scaled to unit variance. *Blue* denotes relative high methylation, *yellow* relative low methylation. **b** Correlation scores of the 67-CpG *HOTAIR* DNAme signature with the corresponding DNAme profiles in embryonic stem cells (*ESC*), reprogrammed MSCs (*iPSC*; induced pluripotent stem cells), ovarian cancer cell lines (*OVC*) with and without stable expression of *HOTAIR* and MSCs harvested at late (more than four passages) or at early passage (fewer than four passages); *P* = 10^−6^ comparing all MSCs to combined ESC/iPSC, *P* = 10^−5^ comparing all OVC to all MSC. **c** Kaplan-Meier curves and log rank test *P* value of carboplatin-treated patients with a high and low DNAme *HOTAIR* signature score (INNSBRUCK set). **d** Validation of the DNA methylation *HOTAIR* signature in an independent large set of carboplatin-treated serous ovarian cancers from TCGA set (*n* = 316). **e** Corresponding Kaplan-Meier curve of *HOTAIR* signature predictions in the non-carboplatin-treated subset of the TCGA set (*n* = 26). **f** Validation of the DNA methylation *HOTAIR* signature in an independent large set of carboplatin-treated ovarian cancers (EUROPE set, *n* = 121). **g** Corresponding Kaplan-Meier curve of *HOTAIR* signature predictions in the non-carboplatin (cisplatin) treated subset (EUROPE set, *n* = 85), validating the specificity of the signature. *HA* HOTAIR, *OS* overall survival

As *HOTAIR* is known to modulate chromatin, in particular at PCGTs, we asked if DNAme differed between *HOTAIR*-expressing and non-expressing ovarian cancer samples. Since *HOTAIR* expression was associated with carboplatin resistance, we restricted the analysis to the 63 carboplatin-treated patients in the INNSNRUCK set. Of these 63 samples, 35 expressed *HOTAIR* whilst 28 did not. We identified a 67-CpG DNAme signature representing a statistically significant association with *HOTAIR* expression. Boxplots of beta methylation values of the 67 CpGs confirmed the relatively large differences in methylation between *HOTAIR* expressors and non-expressors (Additional file 11). We observed that many of the top CpGs mapped to PCGTs (Fig. 2a) and found that PCGTs (defined as PRC2 targets in both human embryonic stem cells and human embryonic fibroblasts, but not PRC2 targets in breast cancer cells) were highly enriched among CpGs hypermethylated in *HOTAIR* expressors (Additional file 12). *HOTAIR* expression was also associated with lower expression of PCGTs, in particular human embryonic fibroblast PCGTs, in the GRONINGEN set [23], supporting the role of *HOTAIR* as an epigenetic regulator of MSCs in ovarian cancer (Additional file 13). By correlating the 67-CpG DNAme signature to the methylation profile of any given sample, a correlation score was obtained which can be viewed as a DNA-based surrogate for *HOTAIR* expression. We first evaluated this correlation score in early passage (multipotent) MSCs, late passage (more differentiated) MSCs, reprogrammed MSCs, embryonic stem cells [29] and ovarian cancer cell lines, demonstrating that the *HOTAIR* DNAme signature is likely to be a surrogate marker for either multipotent MSCs within the ovarian cancer tissue, or for ovarian cancer cells with an increased tendency to undergo EMT (Fig. 2b; Additional file 14).

### The *HOTAIR*-DNAme signature predicts survival outcome

Given the relationship between *HOTAIR* expression and carboplatin resistance, we next checked that the *HOTAIR* DNAme signature would be similarly associated with outcome in the 63 carboplatin-treated subgroup in the INNSBRUCK set. As expected, the score obtained by correlating the sample-specific DNAme profile to the *HOTAIR* DNAme signature was predictive of carboplatin resistance in univariate as well as in multivariate Cox-regression analyses adjusted for age, stage and size of residual tumour (Additional file 15). In order to build a single-sample classifier we also optimized a cutoff on the correlation score of the ovarian cancer tissue samples using ROC analysis to ensure approximately 80 % sensitivity and 80 % specificity between the DNAme signature and *HOTAIR* expression (Additional file 16). Dividing the samples into two groups based on this cutoff further confirmed a significant difference in survival rates of these two subgroups of patients, i.e., samples with a high DNAme signature score had a HR of 3.07 (*P* = 0.001) for death relative to those with a low surrogate score (Fig. 2c).

### Validation of *HOTAIR* DNAme signature in three independent large cohorts

To validate our *HOTAIR* DNAme signature, we first tested it in an independent large data set of serous ovarian cancers from TCGA set, consisting of 316 patients who received carboplatin-based therapy and 26 who received cisplatin. For each of the carboplatin-treated patients, we computed a carboplatin resistance score by correlating the DNAme profile of the tumour to the previously determined 67-CpG DNAme *HOTAIR* signature. This score predicted outcome in both univariate as well as multivariate Cox-regression analyses (Additional file 15), and was a much stronger predictor of outcome than scores constructed using random signatures (Additional file 17). Using the previously determined cutoff to assign samples into high and low DNAme signature score groups further demonstrated the robustness of the predictive classifier in the carboplatin-treated subgroup (Fig. 2d). Of note, the prediction obtained using the *HOTAIR* DNAme signature outperforms the classification obtained using either mRNA or microRNA expression predictors, as reported in TCGA study [24]. In contrast to the carboplatin-treated group, the *HOTAIR* DNAme signature was not predictive of outcome in the 26 patients in TCGA set who did not receive carboplatin (Fig. 2e).

We further tested the *HOTAIR* DNAme signature in an independent set of 121 carboplatin-treated patients (EUROPE set) with DNAme profiles generated using a different assay (Illumina Infinium Human Methylation 450 k). In order to more rigorously assess the specificity of the signature, it was also tested in a further 85 cisplatin-treated patients from the same EUROPE cohort which were also profiled with the same 450 k technology. Once again, the DNAme signature-based surrogate scores for *HOTAIR* expression correctly predicted carboplatin resistance (Fig. 2f; Additional file 18), with no association observed in the cisplatin-treated subgroup (Fig. 2g; Additional file 18).

Further strengthening the robustness of the *HOTAIR* DNAme signature, we found it to be predictive of carboplatin resistance in an additional independent set of 174 carboplatin-treated ovarian cancer patients (ROCHESTER-MAYO set), which had also been profiled with Illumina 450 k DNAme bead arrays (Additional file 19).

### *HOTAIR* expressors respond preferentially to cisplatin-based chemotherapy

In all data sets analysed, we observed a consistent trend for *HOTAIR* expression, or DNAme-based surrogate *HOTAIR* expression, to be associated preferentially with cisplatin response, although statistical significance was not observed in individual data sets. Thus, to investigate this further we used a meta-analysis approach and asked if the type of chemotherapy received was associated with a different response in *HOTAIR* expressors compared with non-expressors (Table 2). In all data sets the risk of death was lower in *HOTAIR* expressors who received cisplatin compared with those who received carboplatin-based therapies, whereas for non-*HOTAIR* expressors the opposite pattern was observed (Table 2; Additional files 20 and 21). Using a combined probability Fisher test in a meta-analysis over all data sets, we found a highly significant interaction between chemotherapy type received and *HOTAIR* expression in dictating response to treatment (*P* < 0.001; Table 2), which was retained in multivariate analysis (*P* = 0.008; Table 2).

**Table 2:** Cox regression analysis of overall survival against chemotherapy received, stratified according to *HOTAIR* positive and negative subgroups

Chemotherapy (set)	*HOTAIR* positive		*HOTAIR* negative		Interaction
	Hazard ratio	*P* value^a^	Hazard ratio	*P* value^a^	*P* value
	(95 % CI)		(95 % CI)		
Cisplatin vs carboplatin (INNSBRUCK)	0.42 (0.23–0.76)	0.003	1.87 (0.95–3.69)	0.068	<0.001
Cisplatin vs carboplatin^b^ (INNSBRUCK)	0.64 (0.33–1.24)	0.187	1.66 (0.69–3.96)	0.255	0.017
Cisplatin vs carboplatin (GRONINGEN)	0.44 (0.18–1.10)	0.071	1.40 (0.49–4.02)	0.525	0.084
Cisplatin vs carboplatin (TCGA)	0.28 (0.04–2.02)	0.18	2.57 (0.33–20.3)	0.35	0.33
Cisplatin vs carboplatin (EUROPE)	0.76 (0.48–1.2)	0.237	1.74 (0.96–3.14)	0.065	0.037
Cisplatin vs carboplatin^b^ (EUROPE)	0.83 (0.52–1.33)	0.436	1.86 (1.02–3.38)	0.042	0.063
Cisplatin vs carboplatin (COMBINED^c^)		0.003		0.076	<0.001
Cisplatin vs carboplatin^b^ (COMBINED^c^)		0.286		0.06	0.008

^a^ *P* values were calculated (in the univariate case) from the Cox-regression likelihood ratio test, while in the multivariate case, the *P* value derives from the Cox-regression Wald test. We note that the Groningen and TCGA sets had only 18 and 26 cisplatin-treated patients, respectively, not allowing for meaningful multivariate results. Interaction was tested by a log-likelihood ratio test between the model with the interaction term (*HOTAIR*:TREATMENT) and the null model without it

^b^ Covariates included stage, grade, age and residual disease whenever these were significant in univariate analysis

^c^ The combined analysis *P* values were derived from Fisher’s combined (meta-analysis) probability test using a chi-square distribution with 8 (2 × 4) degrees of freedom in the univariate case (INNSBRUCK, GRONINGEN, EUROPE, TCGA) and 4 (2 × 2) degrees of freedom in the multivariate case (INNSBRUCK, EUROPE)

### Effect of *HOTAIR* expression and platinum sensitivity in ovarian cancer cell line

**Fig. 3:**
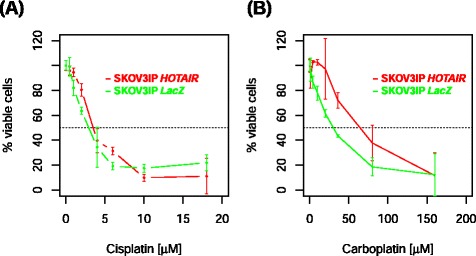
Chemosensitivity of SKOV3IP ovarian cancer cells which are stably transfected with LacZ (control) or *HOTAIR*. Treatment with cisplatin (**a**) and carboplatin (**b**)

In order to test whether *HOTAIR* expression modulates response to carboplatin we used SKOV3IP ovarian cancer cells, which are sensitive to platinum-based chemotherapy and do not express *HOTAIR*. We observed that overexpression of *HOTAIR* in this particular cell line reduces only sensitivity to carboplatin but not cisplatin (Fig. 3). The half maximal inhibitory concentration (IC_50_) shifted from 30 to 60 μM for carboplatin and from 3 to 3.5 μM for cisplatin in LacZ and *HOTAIR*-expressing SKOV3IP cells, respectively. *HOTAIR* expression on two other cell lines had either no effect (A2780) or increased cisplatin sensitivity (OVCAR8) (Additional file 22).

## Discussion

Here we have shown that women with ovarian tumours expressing *HOTAIR* RNA, or an equivalent *HOTAIR*-associated DNAme signature, experience a poorer survival outcome post carboplatin-based chemotherapy compared with *HOTAIR*-ve tumours.

These data offer solid evidence for two novel concepts. First, we demonstrate that not only a non-coding RNA, but importantly also a presumed downstream effect, represented by a specific DNAme signature, reproducibly predicts cancer outcome. This concept is appealing, because (i) a DNA-based biomarker is more stable and (ii) would provide a spatially and temporally more comprehensive surrogate for cancer biology compared with a snap-shot RNA assessment. Second, our data very much challenge the dogma that cisplatin and carboplatin have the same effect on ovarian cancer and that parameters that allow for discrimination between patients that benefit form carboplatin and those that benefit form cisplatin would not exist.

A recent study from Roodhart et al. [9] highlights the complexity of the chemo-resistance response which integrally involves cancer cell non-autonomous factors. The authors used a mouse model and demonstrated that cells with a multilineage differentiation potential, such as embryonic fibroblasts, become activated during treatment with platinum analogs and secrete factors systemically that protect tumour cells against platinum chemotherapeutics. Roodhart et al. [9] demonstrated this for both carboplatin and cisplatin whereas our data — based on 1080 human ovarian cancer samples — demonstrate that *HOTAIR* and its surrogate DNAme signature are associated only with carboplatin resistance and not cisplatin resistance.

Although in the past cisplatin and carboplatin have been considered to be nearly identical drugs and prospective randomized trials showed similar overall survival for cisplatin- and carboplatin-based regimens in ovarian cancer [30, 31], both drugs have substantially different side-effect profiles with higher rates of nausea, vomiting and renal toxicity for cisplatin and thrombocytopenia for carboplatin [32]. Moreover, cisplatin and carboplatin are known to differ in their cell membrane transport characteristics [33]. This is noteworthy because cell membrane transport proteins have been shown to be critical determinants of platinum drug sensitivity/resistance, possibly as a result of secreted factors from MSCs which can affect transport characteristics. Lending further credence to our data, in cervical cancer (a disease which is known to expresses high levels of *HOTAIR* [34]) carboplatin has been repeatedly reported to be a less effective platinum analog than cisplatin [35–37].

Although we provide unprecedented strong evidence for a non-coding RNA and its DNAme surrogate signature to be a predictive and prognostic marker in ovarian cancer, there are several limitations to our study. First, although in situ hybridisation for *HOTAIR* has been successfully established for cell lines, we were not able (despite substantial efforts; data not shown) to determine the specific subset of *HOTAIR*-expressing cells within the bulk tumour sample. Hence, at this stage we can only state that *HOTAIR* expression serves as an excellent surrogate for “mesenchyme-ness” of a cancer, not knowing whether this reflects the presence of MSCs in the tumour stroma or whether it is a reflection of the number of cancer cells that have undergone EMT, or a combination of both. Second, we don’t provide a mechanistic model as to why and how *HOTAIR* modulates carboplatin and cisplatin response differently. Whereas recent evidence shows that *HOTAIR* promotes proliferation by modulating cell cycle and apoptosis [38], no evidence exists to show that this has an impact on platinum resistance. Our findings are consistent with the view that *HOTAIR* modulates the epigenome at the level of the DNA methylome in both cancer cells and tumour stroma. Although still speculative, it is likely that the differential response of carboplatin and cisplatin is due to underlying differences in how the MSC biology of tumour stroma and the EMT characteristics of cancer cells affect the two drugs. Support for this view comes from recent data (unpublished): we have analysed MSCs and modulated expression of *HOTAIR*. Overexpression and knockdown of *HOTAIR* inhibited or stimulated, respectively, in vitro differentiation of MSCs. Modification of *HOTAIR* expression evoked consistent effects on gene expression, particularly in polycomb group target genes and genes involved in cancer. Furthermore, overexpression and knockdown of *HOTAIR* resulted in DNAme changes that are enriched in HOTAIR binding sites.

Despite these limitations, our findings have a number of immediate clinical implications. We provide a solid rationale for prospective randomized clinical trials — ideally in a neo-adjuvant setting — to assess whether the *HOTAIR* DNAme signature is an appropriate tool to stratify women with ovarian cancer (and possibly also other cancers) into groups which benefit preferentially from cisplatin or from carboplatin treatment. Strategies to reduce *HOTAIR* activity (i.e., by intra-peritoneal *HOTAIR* small interfering RNA) may lead to a novel strategy to (re)sensitize cancers to chemotherapy.

## Conclusions

Our data demonstrate that *HOTAIR* and its surrogate DNAme signature play a crucially important role in ovarian cancer biology and provide novel leads to revisit the clinically important field of platinum resistance in this disease.

## Additional files

Additional file 1:
**Additional description of methods and sample sets.** (PDF 221 kb) Additional file 2:
**Clinicopathological characteristics of ovarian cancer patients (INNSBRUCK set) for which** ***HOTAIR*** **expression and DNAme have been done.** (PDF 189 kb) Additional file 3:
**Normalised beta-value DNAme data matrix that was used to identify the 67** ***HOTAIR*** **-associated CpGs.** This data matrix has dimensions 5000 CpGs (the 5000 most variable positions) by 134 samples; samples are annotated according to whether they are *HOTAIR* + ve/-ve and type of chemotherapy received. (XLSX 9331 kb) Additional file 4:
**Clinicopathological features of patients (GRONINGEN set) stratified according to** ***HOTAIR*** **expression.** (PDF 161 kb) Additional file 5:
**Clinicopathological characteristics of ovarian cancer patients from TCGA set.** (PDF 178 kb) Additional file 6:
**Clinicopathological characteristics of ovarian cancer patients from the EUROPE set.** (PDF 177 kb) Additional file 7:
**Ten-fold internal cross-validations to identify an optimal DNAme signature.** *Upper panel* shows the total misclassification error (y-axis) as a function of the shrinkage threshold (x-axis) used. *Lower panel* shows the misclassification error for each phenotype (1 = low *HOTAIR* expression, 2 = high *HOTAIR* expression) as a function of the same shrinkage threshold. The optimal minimal classifier was found at a threshold of approximately 1.47, corresponding to a 67-CpG signature at an estimated false discovery rate (FDR) of approximately 0.17 (not shown). The FDR was estimated using a permutation scheme as implemented in the *pamr R-package*, and the relatively low FDR (only about 17 % of the 67 CpGs are expected to be false positives) demonstrates the presence of a genuine DNAme signal associated with *HOTAIR* expression. (PDF 13 kb) Additional file 8:
**Hazard ratios for relapse and death for patients who received carboplatin treatment (a) and patients who did not receive carboplatin treatment (b) in the original INNSBRUCK set.** (PDF 183 kb) Additional file 9:
**Hazard ratios (HR) for death of patients who received carboplatin-based treatment in the GRONINGEN set and had RNA available to analyze** ***HOTAIR.*** (PDF 138 kb) Additional file 10:
**Kaplan-Meier survival estimates in patients from the BERGEN set who received no chemotherapy (untreated group,** ***n*** **= 9) (a) or carboplatin-based chemotherapy** (***n*** **= 40) (b) and stratified according to** ***HOTAIR*** **expression.** Patients (*n* = 49) treated in Bergen (Norway) and whose cancers had >25 % stroma component were analyzed; 8, 2, 34 and 5 had stage 1, 2, 3 and 4 disease, respectively; 32, 6, 9 and 2 had a serous, mucinous, endometrioid and clear cell cancer, respectively. In the untreated group, significantly more patients had stage 1 disease (44 % versus 10 % in the carboplatin group) and no residual disease after primary surgery (75 % versus 38 % in the carboplatin group). The top tertile expressing samples were deemed as high (positive) *HOTAIR* expressors and compared with low/absent (negative) *HOTAIR* expressors. There is no significant difference between high and low *HOTAIR* expressors with regards to grade, stage or residual disease. Comparing *HOTAIR*-positive with *HOTAIR*-negative patients, the hazard ratio is 2.55 (95 % confidence interval 1.14–5.68), *P* value 0.018. (PDF 244 kb) Additional file 11:
**Boxplots of beta methylation values of the 67 CpGs (INNBSRUCK set) which demonstrate the largest difference between** ***HOTAIR*** **-negative (indicated as “** ***1*** **” and** ***green boxes*** **) and** ***HOTAIR*** **-positive (indicated as “** ***2*** **” and** **red boxes** **) ovarian cancer samples.** (PDF 144 kb) Additional file 12:
**Enrichment odds ratios** (***OR***) **with 95 % confidence intervals for PCGTs according to different definitions among CpGs undergoing significant hyper- and hypomethylation with** ***HOTAIR*** ***(HA)*** **expression (top 500 CpGs) in ovarian cancer samples (INNSBRUCK set).** PCGTs are defined as genes associated with SUZ12, EZH2 and H3K27me3 (*TriplePCGT*) or any one of these factors (*SinglePCGT*) in human embryonic stem cells (Lee et al. [16]) or as the ~850 genes that gain H3K27me3 upon overexpression of *HOTAIR* in MDAMB321 breast cancer cells as described by Gupta et al. [10] (*PRC2-MDAMB231*) or PRC2 targets in human embryonic fibroblast (*PRC2-hEF*) (Bracken et al. 2006). (PDF 5 kb) Additional file 13:
**Expression analyses for the GRONINGEN set. Of the 175 samples from the GRONINGEN set, 114 samples had matched array expression profiles (Operon Human v3 ~ 35 K 70-mer two-color oligonucleotide arrays, GSE13876, Crijns et al.** [23 **]) and** ***HOTAIR*** **expression** **(36** ***HOTAIR*** **-ve, 78** ***HOTAIR +*** **ve).** We correlated the expression of genes on the array to *HOTAIR* expression and selected genes with a t-statistic *P* value < 0.05 (1147 genes). From these we then selected those genes which are PRC2 targets in MDAMB231 breast cancer cell lines (*PRC2-MDAMB231*; Gupta et al. [10]) or in human embryonic fibroblasts (*PRC2-hEF*; Bracken et al. 2006). There were 42 and 97 such PRC2-MDAMB231 and PRC2-hEF genes, respectively. The average expression (*AvExp*) over these enriched PRC2-MDAMB231 and PRC2-hEF genes was then computed for each sample separately and these values are compared between *HOTAIR*-ve and *HOTAIR* + ve samples. Wilcoxon rank sum test *P* value is given. (PDF 5 kb) Additional file 14:
***HOTAIR*** **DNAme score in ovarian cancer cell lines and MSCs depending on number of passages after stable** ***HOTAIR*** **transfection (ovarian cancer cells) or after starting in vitro culture (MSCs).** The *HOTAIR* DNAme score is the Pearson correlation coefficient between the 67-CpG *HOTAIR* DNAme signature and the corresponding DNAme profile of each of the cell lines. Whereas neither *HOTAIR* expression nor passage number had an impact on the correlation coefficient in ovarian cancer cell lines, early passage (multipotent) MSCs (irrespective of whether they had been irradiated or not) showed a much stronger association with the 67-CpG *HOTAIR* signature than higher passage (senescent) MSCs (from the same individuals). (PDF 128 kb) Additional file 15:
**Performance of the DNAme signature in the primary INNSBRUCK carboplatin-treated ovarian cancer set and TCGA set.** (PDF 215 kb) Additional file 16:
**Predicting** ***HOTAIR*** **expression with the 67-CpG DNA methylation signature.** A ROC analysis was used to optimize the cutoff of the correlation score between the 67-CpG DNAme signature and the corresponding DNAme of each of the 63 ovarian cancer samples (INNSBRCUK set, carboplatin-treated subgroup). A correlation score of −0.16 ensures approximately 80 % sensitivity and 80 % specificity between the 67-CpG DNAme signature and *HOTAIR* RNA expression. (PDF 4 kb) Additional file 17:
**Comparison of the predictive score of our** ***HOTAIR*** **DNA methylation signature** (***red***) **to those obtained from 1000 randomized signatures** ***(green)*** **(randomized signatures have the same number of CpGs as the original signature).** To further substantiate our results, we compared the predictive score of our *HOTAIR* DNAme signature to a randomized signature (same number of CpGs as in the original signature) obtained by randomly permuting the methylation profiles of CpGs in TCGA data. By comparing the observed Cox score to the ones obtained by a large number of such randomizations, we evaluated the predictive significance of the *HOTAIR* DNAme signature against the background probability. The randomization procedure showed that in only 2 of the 10,000 runs (*P* < 0.001) the Cox score was more significant than the observed (unpermutated) score. Thus, the selection of predictive CpGs in our primary ovarian cancer set identified CpGs more likely to be predictive of carboplatin response in the independent TCGA data. (PDF 3 kb) Additional file 18:
**Performance of the DNAme signature in the carboplatin (a) and cisplatin (b) treated EUROPE set.** (PDF 212 kb) Additional file 19:
**Performance of the DNAme signature in the carboplatin-treated set from the ROCHESTER-MAYO set.** (PDF 201 kb) Additional file 20:
**Kaplan-Meier survival estimates for patients whose tumours expressed** ***HOTAIR*** **(a, c) and whose tumours did not express** ***HOTAIR*** **(b, d) in the INNSBRUCK (a, b) and GRONINGEN sets (c, d).** Survival analysis was performed according to the type of chemotherapy patients received: carboplatin monotherapy (*Carbo-Mono*), carboplatin-paclitaxel (*Carbo-Pacli*), carboplatin-cyclophosphamide (*Carbo-Cyclo*) or cisplatin-based chemotherapy (*Cisplat*). Hazard ratios (*HR*) and *P* values were calculated comparing cisplatin- with carboplatin-based chemotherapy regimens. (PDF 281 kb) Additional file 21:
**Kaplan-Meier survival estimates in patients in TCGA set whose tumours had a high (a) and a low (b)** ***HOTAIR*** **DNA methylation score.** Survival analysis was performed according to the type of chemotherapy patients received: carboplatin based (*Carboplatin*), carboplatin-cisplatin (*Carbo-Cisp*) or cisplatin chemotherapy (*Cisplatin*). Hazard ratios (*HR*) and *P* values were calculated comparing cisplatin- with carboplatin-based (non-cisplatin-containing) chemotherapy regimens. (PDF 8 kb) Additional file 22:
**Chemosensitivity of A2780 and OVCAR8 ovarian cancer cells which are stably transfected with LacZ (control) or** ***HOTAIR***. Cells were treated with cisplatin (0.5–25 μM) or carboplatin (10–160 μM) for 3 days and analysed by the cell survival MTT assay (Sigma). (PDF 397 kb)

## Abbreviations

CI: confidence interval
ct: cycle threshold
DNAme: DNA methylation
EMT: epithelial–mesenchymal transition
FDR: false discovery rate
GEO: Gene Expression Omnibus
*HOTAIR*: HOX transcript antisense intergenic RNA
HR: hazard ratio
MSC: mesenchymal stem cell
PCGT: polycomb group target
PRC2: polycomb repressor complex 2
ROC: receiver operating characteristic
TCGA: The Cancer Genome Atlas.
