# Supplementary material for: HOTAIR and its surrogate DNA methylation signature indicate carboplatin resistance in ovarian cancer
Source: Genome Med. 2015 Oct 24;7:108. doi: 10.1186/s13073-015-0233-4 (PMC4619324; doi:10.1186/s13073-015-0233-4)
Supplement: Additional file 1: — Additional description of methods and sample sets. (PDF 221 kb) [file 13073_2015_233_MOESM1_ESM.pdf]

Supplementary Information for

HOTAIR AND ITS SURROGATE DNA METHYLATION SIGNATURE INDICATE CARBOPLATIN RESISTANCE IN OVARIAN CANCER

Andrew E. Teschendorff, Shih-Han Lee, Allison Jones, Heidi Fiegl, Marie Kalwa, Wolfgang Wagner, Kantaraja Chindera, Iona Evans, Louis Dubeau, Arturo Orjalo, Hugo Horlings, Lukas Niederreiter, Arthur Kaser, Winnie Yang, Ellen L. Goode, Brooke L. Fridley, Andrew Berchuck, Richard G. Jenner, Els M. J. J. Berns, Elisabeth Wik, Helga B. Salvesen, G. Bea A. Wisman, Ate G. J. van der Zee, Ben Davidson, Claes G. Trope, Sandrina Lambrechts, Ignace Vergote, Hilary Calvert, Ian J. Jacobs and Martin Widschwendter

Supplementary Methods

Sample sets:

We analyzed 6 different sample sets:

(1) Denoted "**INNSBRUCK**": Primary ovarian cancer samples (n=134, 24-87 years, median 62.7 years at diagnosis, Additional data file 2): All patients were treated at the Department of Obstetrics and Gynecology of the Innsbruck Medical University, Austria between January 1989 and February 2000. The study was approved by the ethical committee of the Medical University Innsbruck (reference number: UN4044). Tumor specimens were obtained immediately after surgery and then evaluated by a single pathologist. One part of the tissue was pulverized under cooling with liquid nitrogen and stored at -70°C, and one part was formalin fixed and embedded in paraffin. Of the 134 patients, 34 received single agent Carboplatin, 29 combined Carboplatin and Paclitaxel, 55 combined Cisplatin and Cyclophosphamide, and 16 did not receive chemotherapy (13, 2 and 1 who had FIGO stage I, II and III respectively). After primary treatment, all of the patients were monitored at intervals increasing from 3 months to 1 year until death or the end of the study. Clinicopathological features are shown in Table 1. The median survival time was 3.8 years.

(2) Denoted "**GRONINGEN**": Primary ovarian cancer samples (n=175, 21-83 years, median 60.0 years at diagnosis, Additional data file 4): All patients were treated at the University Medical Center in Groningen, Netherlands between 1990 and 2003 and most of the samples have been described recently. Clinicopathological features are shown in Additional data file 4. The median survival time for all patients in this set which consisted only of stage III/IV patients was 2.1 years.

(3) Denoted "**TCGA**": Primary ovarian cancer samples (n=342, serous ovarian cancers, median 58 years at diagnosis; 316 received Carboplatin-based chemotherapy), analyzed within The Cancer Genome Atlas (TCGA) program, and for which DNAme data were publicly available (24) (Additional data file 5). The median survival time for all patients in this set was 2.6 years.

(4) Denoted **"EUROPE"**: Primary ovarian cancer samples from three European Cancer centres (Leuven, Oslo, Rotterdam; n=206, median 58 years at diagnosis, Additional data file 6). The median survival time for all patients in this set was 3.4 years.

(5) Denoted **"BERGEN"**: Primary ovarian cancer samples from Bergen (n=49): see Additional data file 10.

(6) Denoted **“ROCHESTER-MAYO”**: Primary serous ovarian cancer samples from the Mayo Clinic in Rochester, MN, USA (n=174): see Additional data file 19.

High grade serous ovarian cancer samples (n=15) to assess HOTAIR expression in ISH experiments were provided by the University of Southern California, CA, USA.

<u>*HOTAIR* expression:</u>

Total cellular RNA was extracted from the tumor specimens by the acid guanidium thiocyanate-phenol-chloroform method, as previously described (Widschwendter et al., 2000). Reverse transcription of RNA was performed as previously described (Muller et al, 2003). Primers and probes for *HOTAIR* were designed using Primer Express (Applied Biosystems, Foster City, CA, USA). BLASTN searches were conducted (Gupta et al, 2010) to confirm the total gene specificity of the nucleotide sequences chosen for the primers and probes. To prevent amplification of contaminating genomic DNA, the probe was placed at the junction between exon 4 and 5 [(amplicon location: NR_003716; 421-514). Forward: 5’-TGT AAT TGC TGG TTT AGG TTG CA-3’; Reverse: 5’-CTG GCA GAG AAA AGG CTG AAA T-3’; TaqMan Probe: 5’ FAM-TTC TCT CGC CAA TGT GCA TAC TTA TAA G-3’BHQ1] or exon 2 and 3 [(amplicon location: NR_003716; 216-285). Forward: 5’-TCC CGG AGG TGC TCT CAA T-3’; Reverse: 5’-AGG CTT CTA AAT CCG TTC CAT TC-3’; TaqMan Probe: 5’ FAM-AGA AAG GTC CTG CTC CGC TTC GCA-3’BHQ1]. Primers and probes for the TATA box-binding protein (TBP; a component of the DNA-binding protein complex TFIID; was used as endogenous RNA control) were used according to Bieche et al (1999). Specificity of all reactions for mRNA only was confirmed prior to use. Real-time PCR was performed in triplicates using an ABI Prism 7900HT Detection System (Applied Biosystems, Foster City, CA) as previously described (Muller et al, 2003). The standard curves were generated using serially diluted solutions of standard cDNA derived from the HOC-7 or A2780 ovarian carcinoma cell-line. The mean (standard deviation) for TBP Ct values was 27.2 (1.2) for the Innsbruck set and 26.5 (1.2) for the Groningen set. Samples in which *HOTAIR* was not amplified by real-time PCR after 45 cycles were classified as *HOTAIR*-ve.

<u>Ovarian cancer cell line experiments and stable expression of *HOTAIR*:</u>

We analysed SKOV3IP, OVCAR8 and A2780 ovarian cancer cell lines which were maintained in RPMI media (Sigma) with 10% FBS (Appleton Woods). The *HOTAIR* and LacZ constructs were kindly provided by Dr. Chang (Stanford) (Gupta et al, 2010). Retroviral particles were produced by transfection of HEK293T cells with 1.5 µg each of *HOTAIR*-LZRS and LacZ plasmids along with 1 µg each of MLV gag-pol expression plasmid pCMVi and VSV-G expression plasmid pMDG. Cell lines were infected and *HOTAIR*/LacZ

overexpressing cells were obtained by single cell cloning. The *HOTAIR* expression of each cell clone was determined by real-time PCR. The LacZ expression was examined by X-Gal staining. DNAme of LacZ and HOTAIR transfected cells was analysed after various passages in cell culture.

Detailed Statistics:

To test for differences in categorical variables, we used the Chi square test. Impact of *HOTAIR* expression on ovarian cancer survival was ascertained using log rank test and Kaplan Meier curves. To demonstrate the effect of *HOTAIR* expression on DNAme patterns, we first performed univariate analysis to rank CpGs according to their association with *HOTAIR* expression. Since DNAme data is not normally distributed, we performed this analysis in two distinct ways: (i) using the non-parametric Wilcox-test P-value to rank CpGs and Somers rank correlation (Dxy) to identify CpGs hypermethylated or hypomethylated in *HOTAIR* expressors, (ii) using the parametric t-test. Both algorithms yielded very similar rankings (very strong overlap of top ranked CpGs), and since the t-test identified CpGs with larger effect sizes, we adopted a parametric approach to derive a *HOTAIR* associated DNAme signature. Specifically, we first identified the 5000 most variable CpGs and then used the shrunken centroids method (Prediction Analysis for Microarrays in R (PAMR)) (Tibshirani et al, 2002) to (i) rank the selected CpGs according to differential methylation between *HOTAIR* expressors and non-expressors and (ii) to also derive a corresponding DNAme signature. We used ten-fold internal cross-validations to identify an optimal DNAme signature composed of the top 67 PAMR ranked CpGs (Additional data file 7) at an estimated false discovery rate (FDR) of approximately 0.17. The FDR was estimated using a permutation scheme and the relatively low FDR demonstrated the presence of a genuine DNAme signal associated with *HOTAIR* expression. For PCGT enrichment analysis we relaxed the threshold to include the top 500 ranked CpGs (FDR<0.3), and divided the 500 into the 233 which were hypermethylated and the 267 which were hypomethylated in high *HOTAIR* expressors. To construct a single-sample DNAme- based classifier that could unambiguously assign any given sample to a low or high *HOTAIR* expression group, we first computed correlation scores (Pearson correlation) between the 67 CpG signature and the DNAme profile of each sample in the training set ("INNSBRUCK" Set). An optimal cut-off (value=-0.16) in these correlation scores was then determined by ROC analysis to ensure at least 80% sensitivity and specificity in predicting *HOTAIR* expression with the "surrogate" 67 CpG DNAme signature.

Validation Analysis: For each sample from the independent cohorts, we computed the correlation between the 67 CpG DNAme signature and its DNAme profile and this correlation

score was then used in Cox-regressions to determine the association between the signature and outcome. Assignment of samples into surrogate high/low *HOTAIR* expression groups was determined using the previously determined cut-off value of -0.16 (from the training set), i.e. samples with a correlation score between the 67 CpG DNAme signature and its DNAme profile less than -0.16 were assigned into the surrogate low-expressor group, with the rest assigned to the surrogate high-expressor group.

Supplementary References

Bieche I, Onody P, Laurendeau I et al. Real-time reverse transcription-PCR assay for future management of ERBB2-based clinical applications. Clin Chem 1999; 45(8 Pt 1):1148-1156.

Bracken AP, Dietrich N, Pasini D et al. Genome-wide mapping of Polycomb target genes unravels their roles in cell fate transitions. Genes & Development 2006, 20 (9),1123.

Crijns AP, Fehrmann RS, de Jong S et al. Survival-related profile, pathways, and transcription factors in ovarian cancer. PLoS Med 2009; 6(2):e24.

Gregory CA, Gunn WG, Peister A and Prockop DJ. An Alizarin red-based assay of mineralization by adherent cells in culture: comparison with cetylpyridinium chloride extraction. Anal. Biochem. 2004; 329: 77-84.

Gupta RA, Shah N, Wang KC et al. Long non-coding RNA HOTAIR reprograms chromatin state to promote cancer metastasis. Nature 2010; 464(7291):1071-1076.

Koch CM, Reck K, Shao K et al.: Pluripotent stem cells escape from senescence-associated DNA methylation changes. *Genome Res* 2013;23:248-259.

Lee TI, Jenner RG, Boyer LA et al. Control of developmental regulators by Polycomb in human embryonic stem cells. Cell 2006; 125(2):301-313.

Muller HM, Fiegl H, Goebel G et al. MeCP2 and MBD2 expression in human neoplastic and non-neoplastic breast tissue and its association with oestrogen receptor status. Br J Cancer 2003; 89(10):1934-1939.

Pittenger MF, Mackay AM, Beck SC, Jaiswal RK, Douglas R, Mosca JD, Moorman MA, Simonetti DW, Craig S, Marshak DR. Multilineage potential of adult human mesenchymal stem cells. Science 1999; 284:143-147.

Schellenberg A, Stiehl T, Horn P, Joussen S, Pallua N, Ho A, Wagner W. Population Dynamics of Mesenchymal Stromal Cells during Culture Expansion. Cytotherapy 2012; 14: 401-411.

Tibshirani R, Hastie T, Narasimhan B, Chu G. Diagnosis of multiple cancer types by shrunken centroids of gene expression. Proc Natl Acad Sci U S A 2002; 99(10):6567-6572

Widschwendter M, Berger J, Hermann M et al. Methylation and silencing of the retinoic acid receptor-beta2 gene in breast cancer. J Natl Cancer Inst 2000; 92(10):826-832.
