# Supplementary material for: HOTAIR and its surrogate DNA methylation signature indicate carboplatin resistance in ovarian cancer
Source: Genome Med. 2015 Oct 24;7:108. doi: 10.1186/s13073-015-0233-4 (PMC4619324; doi:10.1186/s13073-015-0233-4)
Supplement: Additional file 2: — Clinicopathological characteristics of ovarian cancer patients (INNSBRUCK set) for which HOTAIR expression and DNAme have been done. (PDF 189 kb) [file 13073_2015_233_MOESM2_ESM.pdf]

Additional data file 2. Clinicopathological characteristics of ovarian cancer patients ("INNSBRUCK") for which *HOTAIR* expression and DNAme has been done (n=134). Patients are stratified into patients who did not receive Carboplatin (16 did not receive chemotherapy and 55 received Cisplatin/Cyclophosphamide based chemotherapy) and who did receive Carboplatin-based chemotherapy (34 single agent and 29 in combination with Paclitaxel)

**Characteristics**	**n**	**Chemotherapy treatment**		**P**
		**no Carboplatin** (n = 71)	**Carboplatin** (n = 63)	
**Age**				
≤ 62.7 yrs. (median age)	67	40	27	0.119
> 62.7 yrs. (median age)	67	31	36	
**FIGO**				
I/II	37	23	14	0.189
III/IV	97	48	49	
**Tumor grade**				
I/II	78	46	32	0.134
III	51	24	27	
Unknown	5	1	4	
**Histology**				
serous cancer	56	36	20	0.210
mucinous cancer	43	22	21	
endometrioid cancer	24	8	16	
clear cell cancer	6	3	3	
Other	2	1	1	
Fallopian tube cancer	3	1	2	
**Residual disease after surgery**				
no residual disease	46	29	17	0.351
residual disease ≤ 2cm	36	16	20	
residual disease > 2cm	45	22	23	
Unknown	7	4	3	
***HOTAIR* expression**				
Yes	72	37	35	0.731
No	62	34	28	
**Health status***				
no relapse	56	31	25	0.641

Relapse	78	40	38	
**Survival status***				
Alive	39	22	17	0.611
Dead	95	49	46	

Note: The significance level (P) was determined by Chi square test.
* for the entire follow up period.
