# Supplementary material for: HOTAIR and its surrogate DNA methylation signature indicate carboplatin resistance in ovarian cancer
Source: Genome Med. 2015 Oct 24;7:108. doi: 10.1186/s13073-015-0233-4 (PMC4619324; doi:10.1186/s13073-015-0233-4)
Supplement: Additional file 4: — Clinicopathological features of patients (GRONINGEN set) stratified according to HOTAIR expression. (PDF 161 kb) [file 13073_2015_233_MOESM4_ESM.pdf]

Additional data file 4. Clinicopathological features of patients ("GRONINGEN") stratified according to *HOTAIR* expression.

Characteristics		*HOTAIR* RNA expression		P Value*
		**negative** (n = 58)	**positive** (n = 117)	
**Age**				0.262
≤ 62.7 yrs. (median age)	87	25	62	
> 62.7 yrs. (median age)	88	33	55	
**FIGO**				0.665
III	147	50	97	
IV	28	8	20	
**Tumor grade**				0.483
I/II	59	17	42	
III	97	34	63	
Unknown	19	7	12	
**Histology**				0.988
serous cancer	118	39	79	
mucinous cancer	15	5	10	
endometrioid cancer	14	5	9	
clear cell cancer	8	2	6	
Others	20	7	13	
**Residual disease after surgery**				0.161
no residual disease	29	8	21	
residual disease ≤ 2cm	38	18	20	
residual disease > 2cm	97	31	66	
Unknown	11	1	10	
**Chemotherapy****				0.999
Cisplatin based	18	6	12	
Carboplatin based	157	52	105	
**Survival status (5 years)**				0.066
Alive	61	26	35	
Dead	114	32	82	

* P values were calculated with the use of the Chi square test.

** 98 and 59 patients received Carboplatin in combination with Cyclophosphamide and Paclitaxel, respectively; 10 and 8 patients received Cisplatin in combination with Cyclophosphamide and Paclitaxel, respectively.
