# Supplementary material for: HOTAIR and its surrogate DNA methylation signature indicate carboplatin resistance in ovarian cancer
Source: Genome Med. 2015 Oct 24;7:108. doi: 10.1186/s13073-015-0233-4 (PMC4619324; doi:10.1186/s13073-015-0233-4)
Supplement: Additional file 5: — Clinicopathological characteristics of ovarian cancer patients from TCGA set. (PDF 178 kb) [file 13073_2015_233_MOESM5_ESM.pdf]

Additional data file 5. Clinicopathological characteristics of ovarian cancer patients ("TCGA"). Patients are stratified into patients who did receive Cisplatin-based chemotherapy (n=26) or received Carboplatin (Carboplatin-Monotherapy or combination Carboplatin/Cisplatin; n=316).

Characteristics	n	Chemotherapy treatment		P
		**Cisplatin** (n = 26)	**Carboplatin** (n = 316)	
**Age**				0.535
≤ 57 yrs. (median age)	148	14	134	
> 57 yrs. (median age)	152	11	141	
Unknown	42			
**FIGO**				1.000
II	15	1	14	
III/IV	326	25	301	
Unknown	1			
**Tumor grade**				0.091
I/II	33	0	33	
III	301	26	275	
Unknown	8			
**Histology**				NA
serous cancer	341	26	315	
Unknown	1			
**Residual disease after surgery**				0.006
no residual disease	58	10	48	
residual disease ≤ 2cm	189	11	178	
residual disease > 2cm	60	2	58	
Unknown	35			
***HOTAIR* DNAme**				1.000
High	224	17	207	
Low	118	9	109	
**Survival status**				
Alive	217	24	193	0.001
Dead	124	2	122	
Unknown	1			

Note: The significance level (P) was determined by Chi square test.
