# Supplementary material for: HOTAIR and its surrogate DNA methylation signature indicate carboplatin resistance in ovarian cancer
Source: Genome Med. 2015 Oct 24;7:108. doi: 10.1186/s13073-015-0233-4 (PMC4619324; doi:10.1186/s13073-015-0233-4)
Supplement: Additional file 6: — Clinicopathological characteristics of ovarian cancer patients from the EUROPE set. (PDF 177 kb) [file 13073_2015_233_MOESM6_ESM.pdf]

**Additional data file 6. Clinicopathological characteristics of ovarian cancer patients ("EUROPE").** Patients are stratified into patients who did receive Cisplatin-based chemotherapy (n=85) or received Carboplatin-based chemotherapy (n=121).

| **Characteristics** | **n** | **Chemotherapy treatment** **Cisplatin** (n = 85) | **Carboplatin** (n = 121) | **P** |
|---|---|---|---|---|
| **Age** | | | | 0.001 |
| ≤ 57 yrs. (median age) | 97 | 54 | 43 | |
| > 57 yrs. (median age) | 109 | 31 | 78 | |
| **FIGO** | | | | 0.431 |
| II | 31 | 15 | 16 | |
| III/IV | 175 | 70 | 105 | |
| **Tumor grade** | | | | 0.045 |
| I/II | 63 | 33 | 30 | |
| III | 143 | 52 | 91 | |
| **Histology** | | | | 0.001 |
| serous cancer | 165 | 61 | 104 | |
| mucinous cancer | 6 | 3 | 3 | |
| endometrioid cancer | 15 | 4 | 11 | |
| clear cell cancer | 13 | 11 | 2 | |
| undifferentiated | 7 | 6 | 1 | |
| **Residual disease after surgery** | | | | 0.044 |
| no residual disease | | 43 | 79 | |
| residual disease present | | 42 | 42 | |
| ***HOTAIR* DNAme** | | | | 0.774 |
| High | 124 | 50 | 74 | |
| Low | 82 | 35 | 47 | |
| **Survival status*** | | | | |
| Alive | 81 | 33 | 48 | 1.000 |
| Dead | 124 | 51 | 73 | |
| Unknown | 1 | | | |

*Note*: The significance level (P) was determined by Chi square test.
* the log-rank P value (Kaplan Meier statistics; comparing 5 year survival between Carboplatin and Cisplatin treated patients) = 0.900.
