# Supplementary figures and images for: HOTAIR and its surrogate DNA methylation signature indicate carboplatin resistance in ovarian cancer
Source: Genome Med. 2015 Oct 24;7:108. doi: 10.1186/s13073-015-0233-4 (PMC4619324; doi:10.1186/s13073-015-0233-4)

# Number of genes

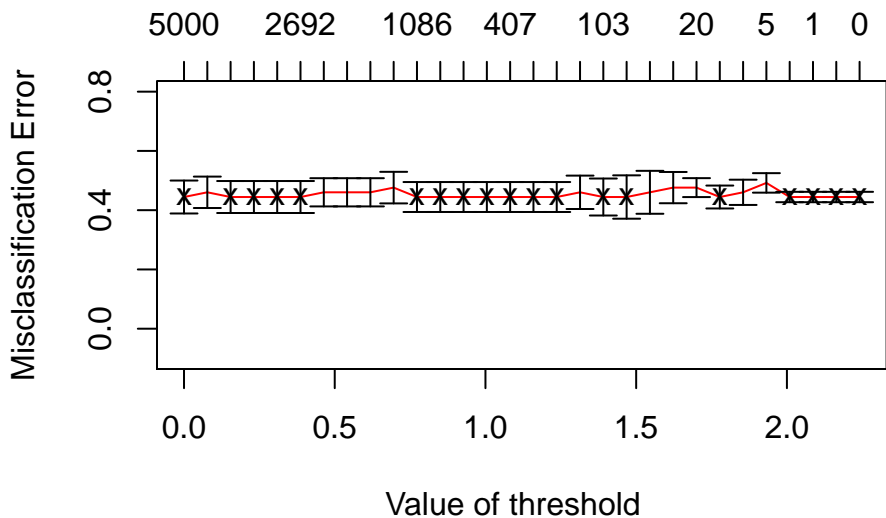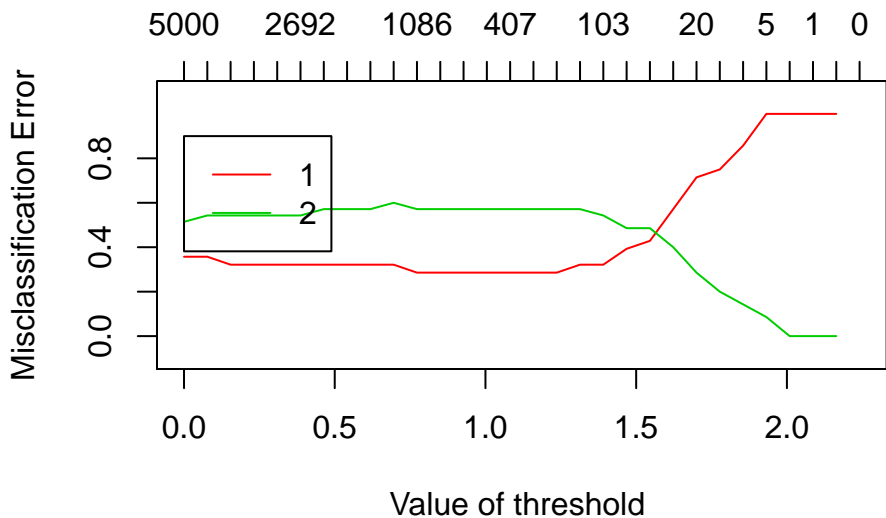

Supplement: Additional file 7: — Ten-fold internal cross-validations to identify an optimal DNAme signature. Upper panel shows the total misclassification error (y-axis) as a function of the shrinkage threshold (x-axis) used. Lower panel shows the misclassification error for each phenotype (1 = low HOTAIR expression, 2 = high HOTAIR expression) as a function of the same shrinkage threshold. The optimal minimal classifier was found at a threshold of approximately 1.47, corresponding to a 67-CpG signature at an estimated false discovery rate (FDR) of approximately 0.17 (not shown). The FDR was estimated using a permutation scheme as implemented in the pamr R-package, and the relatively low FDR (only about 17 % of the 67 CpGs are expected to be false positives) demonstrates the presence of a genuine DNAme signal associated with HOTAIR expression. (PDF 13 kb) [file 13073_2015_233_MOESM7_ESM.pdf]

**(A)**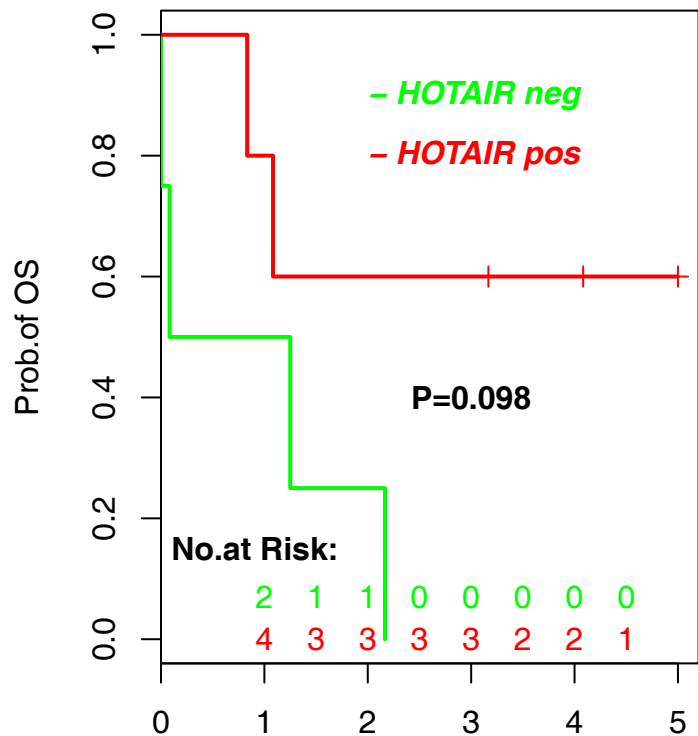**(B)**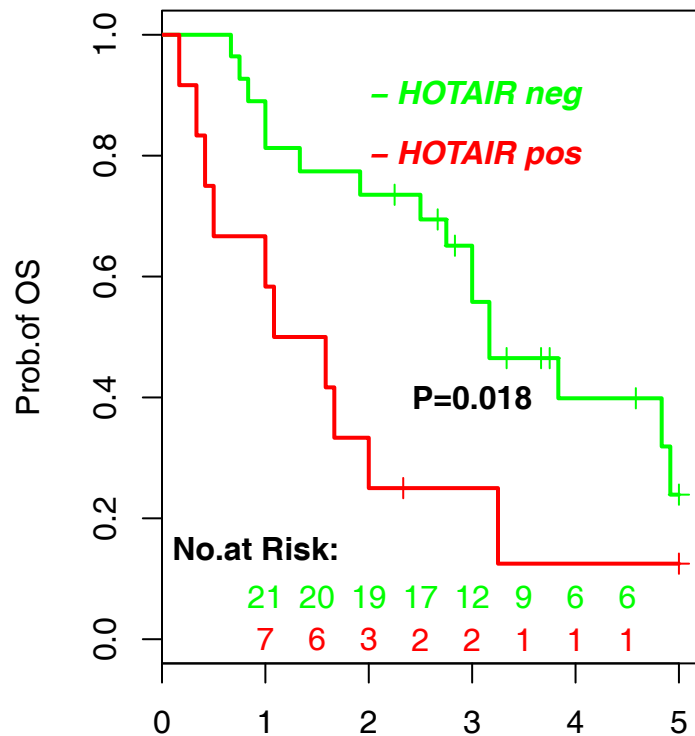

Supplement: Additional file 10: — Kaplan-Meier survival estimates in patients from the BERGEN set who received no chemotherapy (untreated group, n = 9) (a) or carboplatin-based chemotherapy (n = 40) (b) and stratified according to HOTAIR expression. Patients (n = 49) treated in Bergen (Norway) and whose cancers had >25 % stroma component were analyzed; 8, 2, 34 and 5 had stage 1, 2, 3 and 4 disease, respectively; 32, 6, 9 and 2 had a serous, mucinous, endometrioid and clear cell cancer, respectively. In the untreated group, significantly more patients had stage 1 disease (44 % versus 10 % in the carboplatin group) and no residual disease after primary surgery (75 % versus 38 % in the carboplatin group). The top tertile expressing samples were deemed as high (positive) HOTAIR expressors and compared with low/absent (negative) HOTAIR expressors. There is no significant difference between high and low HOTAIR expressors with regards to grade, stage or residual disease. Comparing HOTAIR-positive with HOTAIR-negative patients, the hazard ratio is 2.55 (95 % confidence interval 1.14–5.68), P value 0.018. (PDF 244 kb) [file 13073_2015_233_MOESM10_ESM.pdf]

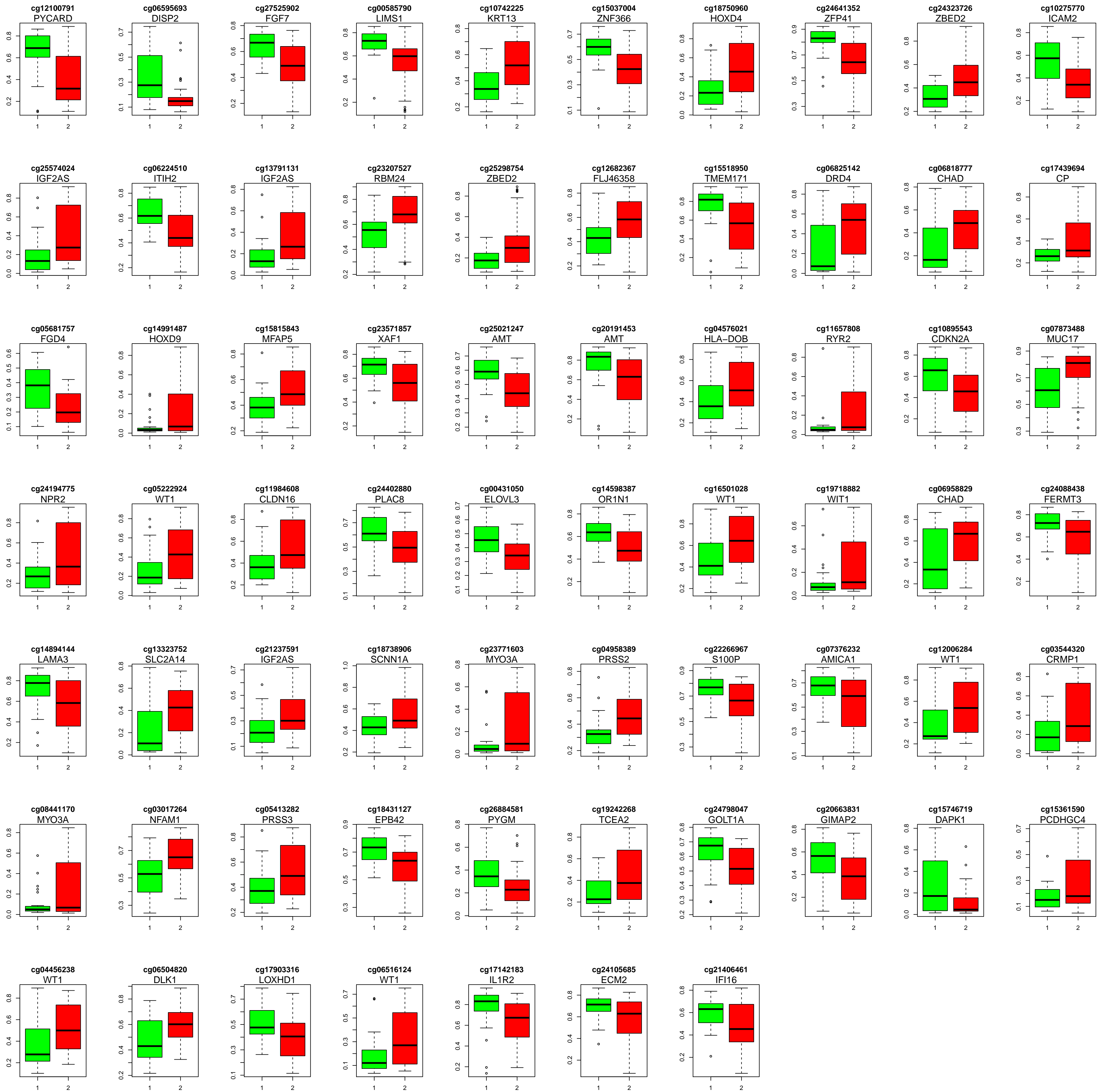

Supplement: Additional file 11: — Boxplots of beta methylation values of the 67 CpGs (INNBSRUCK set) which demonstrate the largest difference between HOTAIR -negative (indicated as “ 1 ” and green boxes ) and HOTAIR -positive (indicated as “ 2 ” and red boxes ) ovarian cancer samples. (PDF 144 kb) [file 13073_2015_233_MOESM11_ESM.pdf]

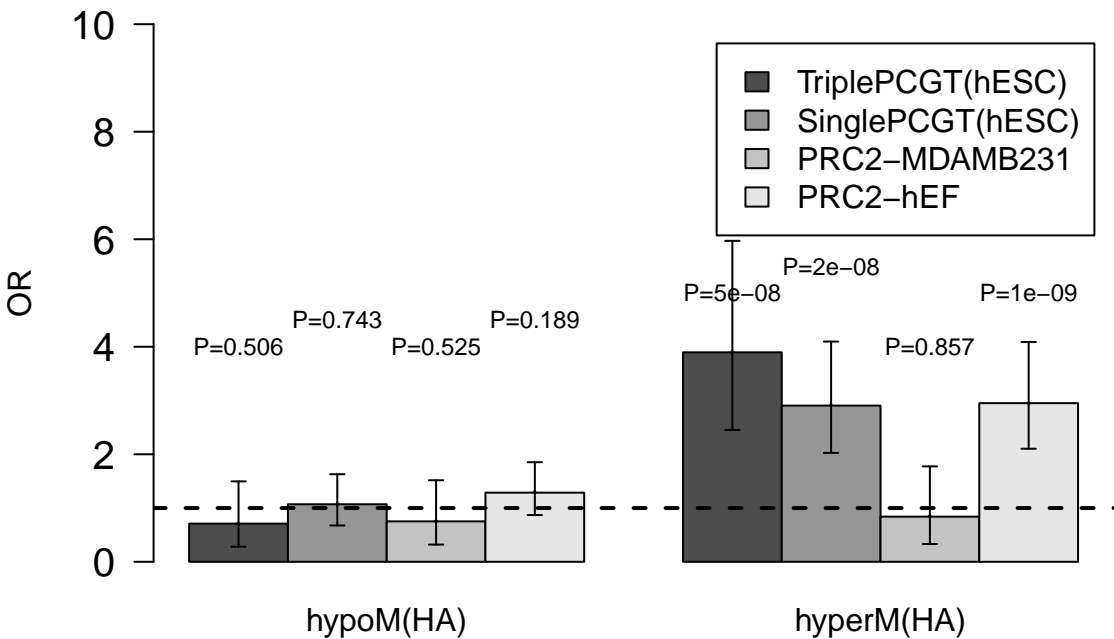

Supplement: Additional file 12: — Enrichment odds ratios (OR) with 95 % confidence intervals for PCGTs according to different definitions among CpGs undergoing significant hyper- and hypomethylation with HOTAIR (HA) expression (top 500 CpGs) in ovarian cancer samples (INNSBRUCK set). PCGTs are defined as genes associated with SUZ12, EZH2 and H3K27me3 (TriplePCGT) or any one of these factors (SinglePCGT) in human embryonic stem cells (Lee et al. [16]) or as the ~850 genes that gain H3K27me3 upon overexpression of HOTAIR in MDAMB321 breast cancer cells as described by Gupta et al. [10] (PRC2-MDAMB231) or PRC2 targets in human embryonic fibroblast (PRC2-hEF) (Bracken et al. 2006). (PDF 5 kb) [file 13073_2015_233_MOESM12_ESM.pdf]

## PRC2-MDAMB231

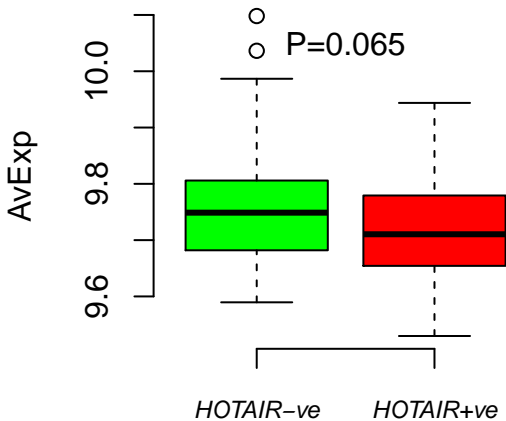

## PRC2-hEF

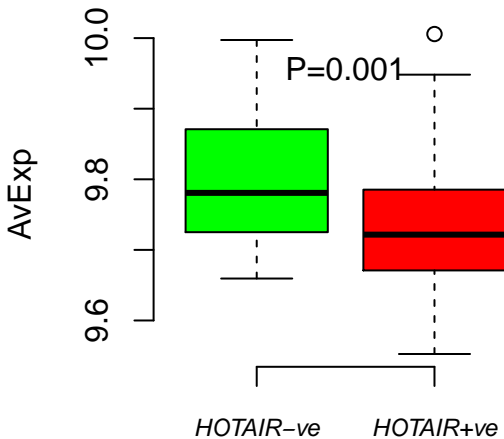

Supplement: Additional file 13: — Expression analyses for the GRONINGEN set. Of the 175 samples from the GRONINGEN set, 114 samples had matched array expression profiles (Operon Human v3 ~ 35 K 70-mer two-color oligonucleotide arrays, GSE13876, Crijns et al. [23 ]) and HOTAIR expression (36 HOTAIR -ve, 78 HOTAIR + ve). We correlated the expression of genes on the array to HOTAIR expression and selected genes with a t-statistic P value < 0.05 (1147 genes). From these we then selected those genes which are PRC2 targets in MDAMB231 breast cancer cell lines (PRC2-MDAMB231; Gupta et al. [10]) or in human embryonic fibroblasts (PRC2-hEF; Bracken et al. 2006). There were 42 and 97 such PRC2-MDAMB231 and PRC2-hEF genes, respectively. The average expression (AvExp) over these enriched PRC2-MDAMB231 and PRC2-hEF genes was then computed for each sample separately and these values are compared between HOTAIR-ve and HOTAIR + ve samples. Wilcoxon rank sum test P value is given. (PDF 5 kb) [file 13073_2015_233_MOESM13_ESM.pdf]

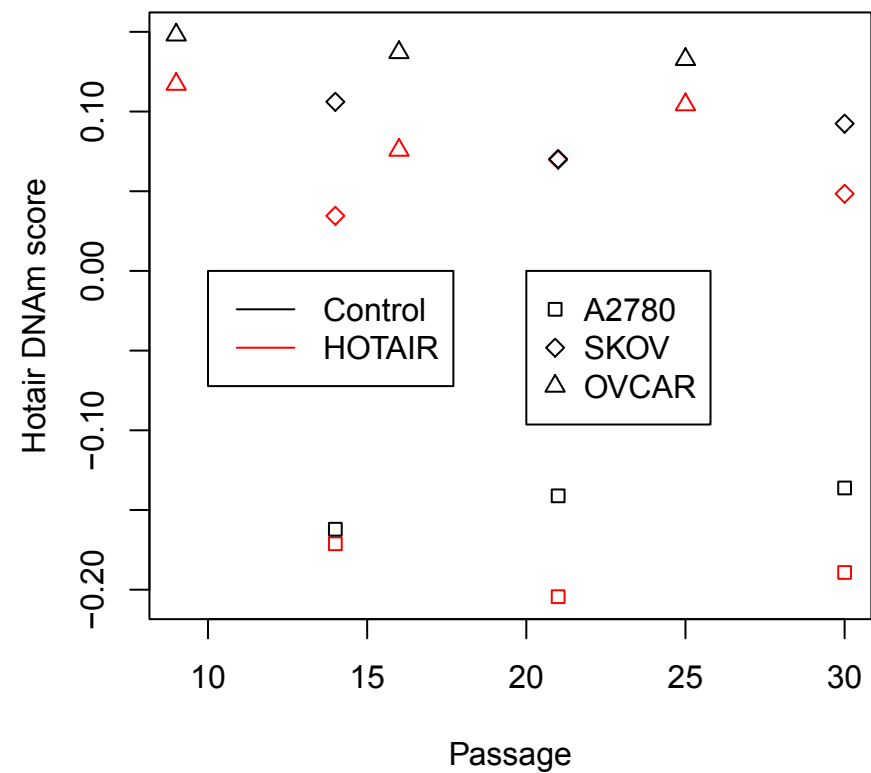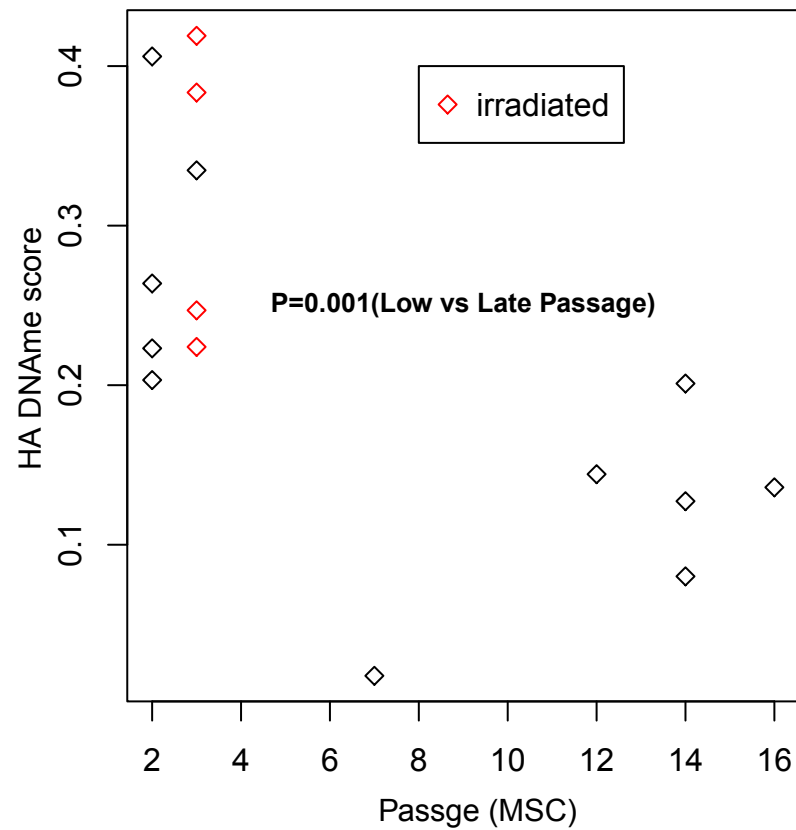

Supplement: Additional file 14: — HOTAIR DNAme score in ovarian cancer cell lines and MSCs depending on number of passages after stable HOTAIR transfection (ovarian cancer cells) or after starting in vitro culture (MSCs). The HOTAIR DNAme score is the Pearson correlation coefficient between the 67-CpG HOTAIR DNAme signature and the corresponding DNAme profile of each of the cell lines. Whereas neither HOTAIR expression nor passage number had an impact on the correlation coefficient in ovarian cancer cell lines, early passage (multipotent) MSCs (irrespective of whether they had been irradiated or not) showed a much stronger association with the 67-CpG HOTAIR signature than higher passage (senescent) MSCs (from the same individuals). (PDF 128 kb) [file 13073_2015_233_MOESM14_ESM.pdf]

## Predicting HA expression with DNAm signature

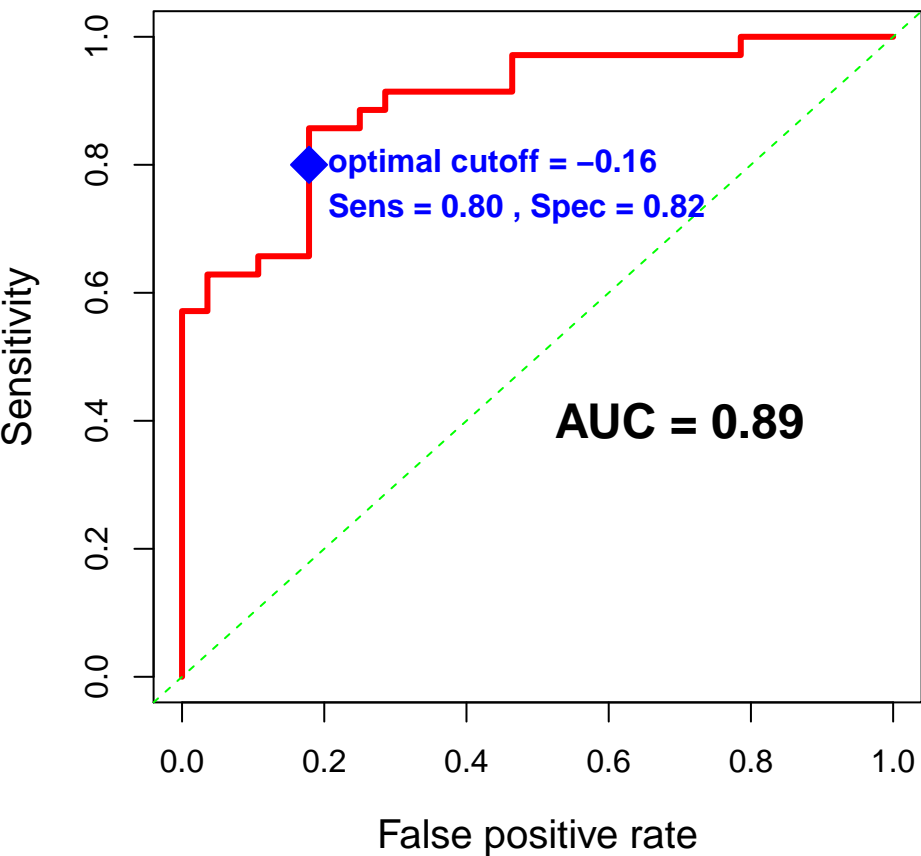

Supplement: Additional file 16: — Predicting HOTAIR expression with the 67-CpG DNA methylation signature. A ROC analysis was used to optimize the cutoff of the correlation score between the 67-CpG DNAme signature and the corresponding DNAme of each of the 63 ovarian cancer samples (INNSBRCUK set, carboplatin-treated subgroup). A correlation score of −0.16 ensures approximately 80 % sensitivity and 80 % specificity between the 67-CpG DNAme signature and HOTAIR RNA expression. (PDF 4 kb) [file 13073_2015_233_MOESM16_ESM.pdf]

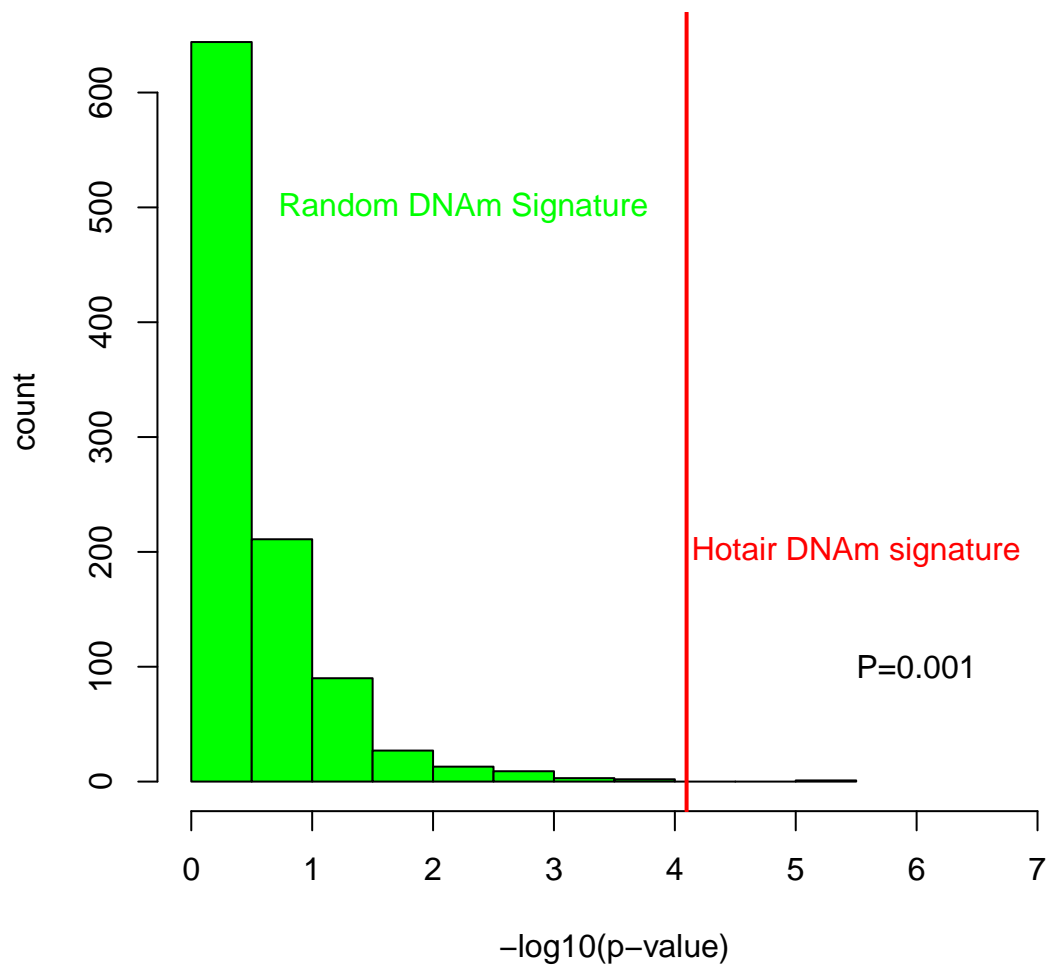

Supplement: Additional file 17: — Comparison of the predictive score of our HOTAIR DNA methylation signature (red) to those obtained from 1000 randomized signatures (green) (randomized signatures have the same number of CpGs as the original signature). To further substantiate our results, we compared the predictive score of our HOTAIR DNAme signature to a randomized signature (same number of CpGs as in the original signature) obtained by randomly permuting the methylation profiles of CpGs in TCGA data. By comparing the observed Cox score to the ones obtained by a large number of such randomizations, we evaluated the predictive significance of the HOTAIR DNAme signature against the background probability. The randomization procedure showed that in only 2 of the 10,000 runs (P < 0.001) the Cox score was more significant than the observed (unpermutated) score. Thus, the selection of predictive CpGs in our primary ovarian cancer set identified CpGs more likely to be predictive of carboplatin response in the independent TCGA data. (PDF 3 kb) [file 13073_2015_233_MOESM17_ESM.pdf]

**(A)** *HOTAIR* expression

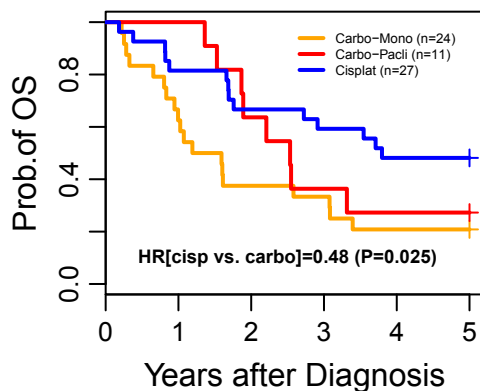

**(B)** *NO HOTAIR* expression

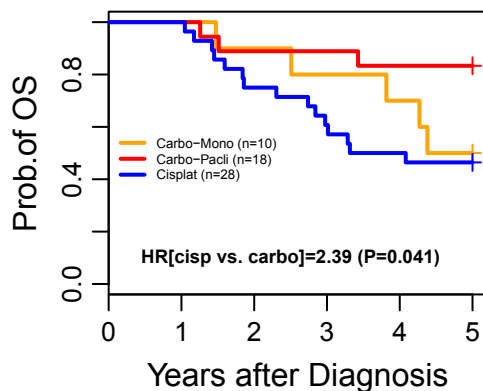

**(C)** *HOTAIR* expression

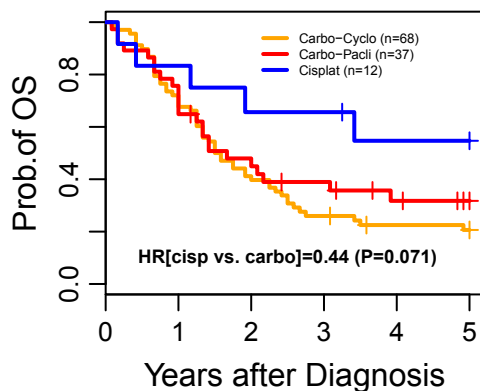

**(D)** *NO HOTAIR* expression

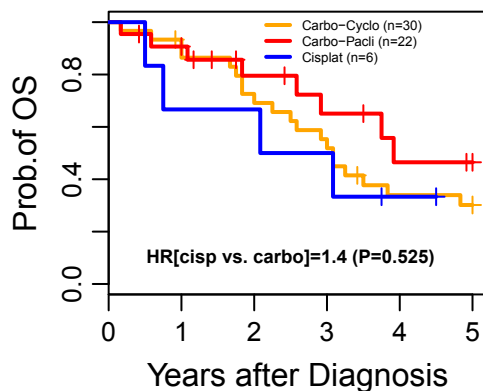

Supplement: Additional file 20: — Kaplan-Meier survival estimates for patients whose tumours expressed HOTAIR (a, c) and whose tumours did not express HOTAIR (b, d) in the INNSBRUCK (a, b) and GRONINGEN sets (c, d). Survival analysis was performed according to the type of chemotherapy patients received: carboplatin monotherapy (Carbo-Mono), carboplatin-paclitaxel (Carbo-Pacli), carboplatin-cyclophosphamide (Carbo-Cyclo) or cisplatin-based chemotherapy (Cisplat). Hazard ratios (HR) and P values were calculated comparing cisplatin- with carboplatin-based chemotherapy regimens. (PDF 281 kb) [file 13073_2015_233_MOESM20_ESM.pdf]

*High HOTAIR DNAm score*

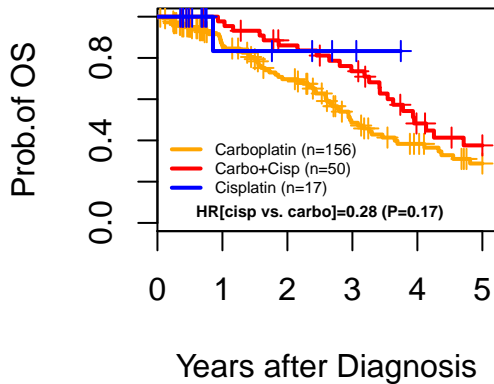

*Low HOTAIR DNAm score*

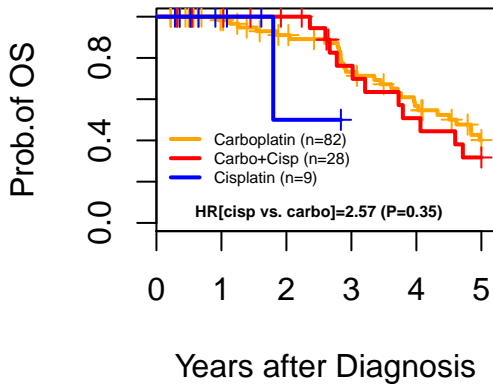

Supplement: Additional file 21: — Kaplan-Meier survival estimates in patients in TCGA set whose tumours had a high (a) and a low (b) HOTAIR DNA methylation score. Survival analysis was performed according to the type of chemotherapy patients received: carboplatin based (Carboplatin), carboplatin-cisplatin (Carbo-Cisp) or cisplatin chemotherapy (Cisplatin). Hazard ratios (HR) and P values were calculated comparing cisplatin- with carboplatin-based (non-cisplatin-containing) chemotherapy regimens. (PDF 8 kb) [file 13073_2015_233_MOESM21_ESM.pdf]

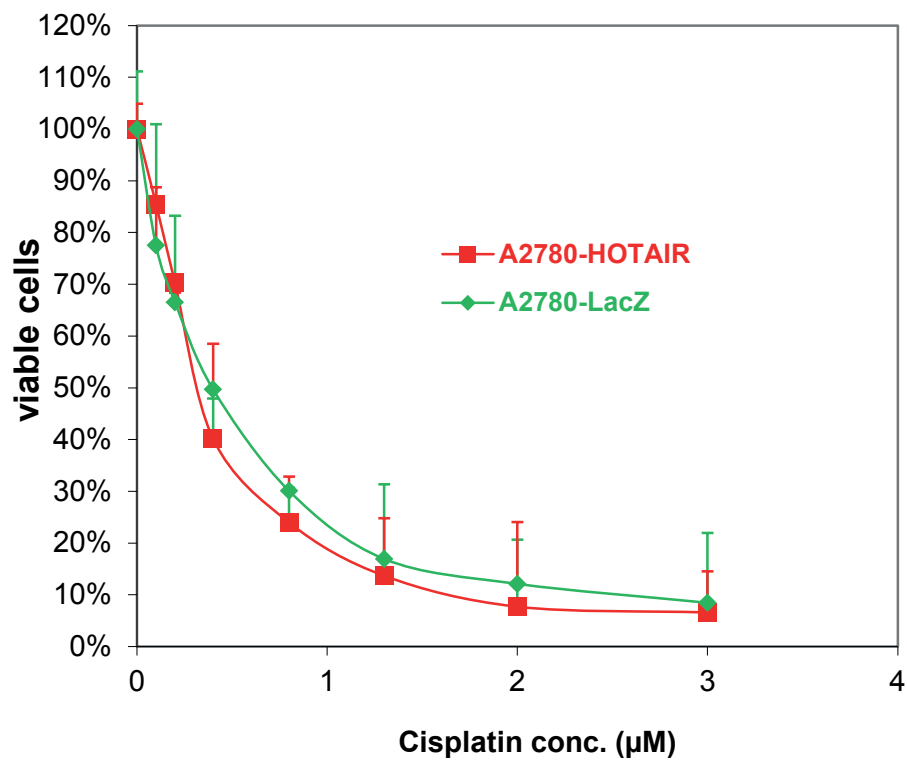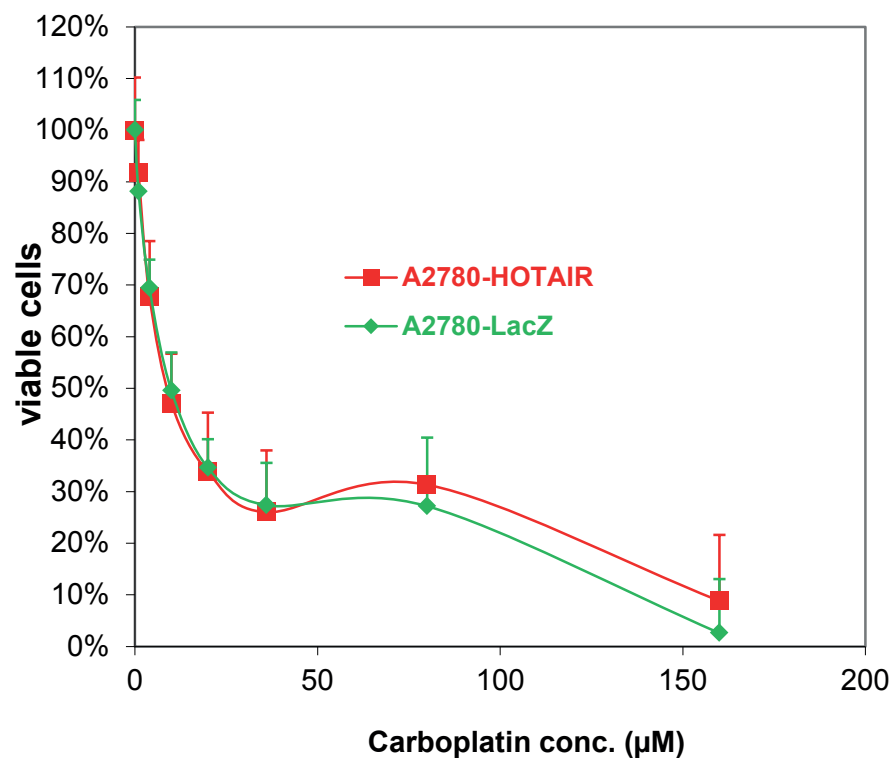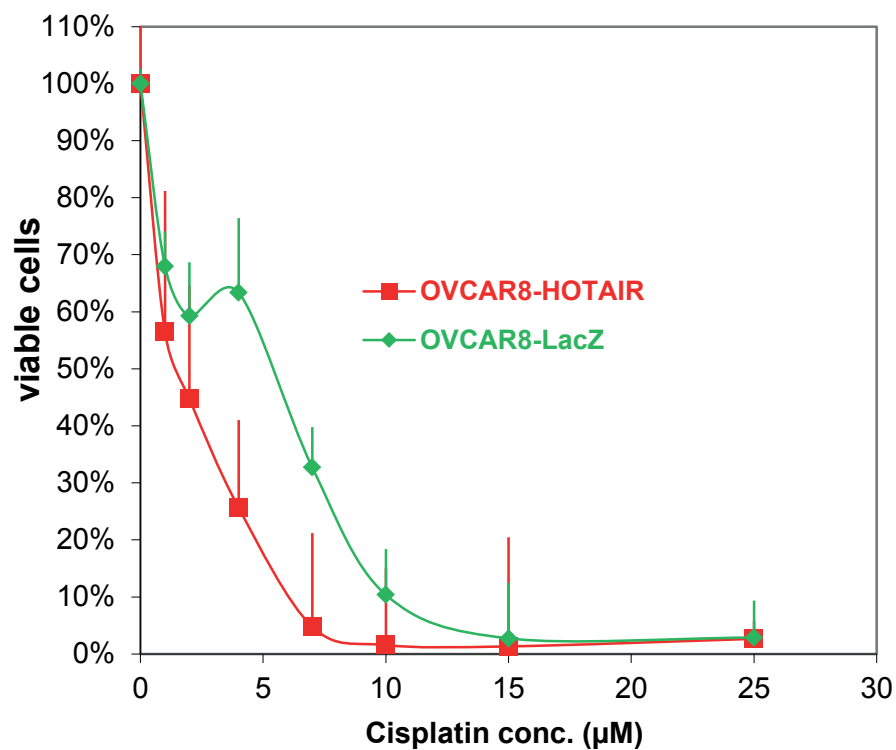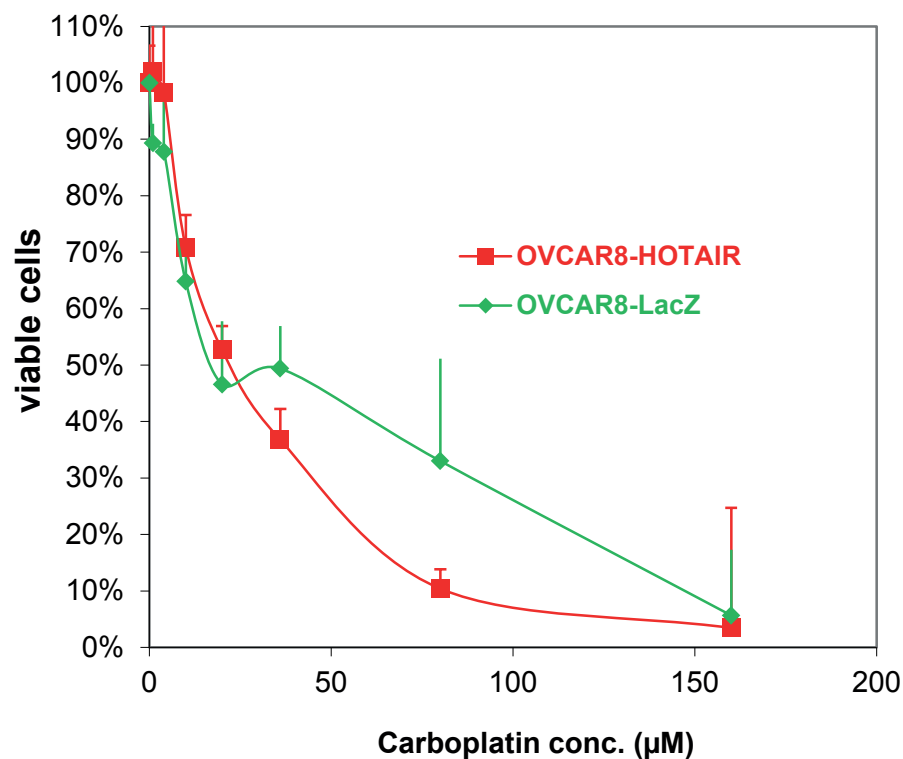

Supplement: Additional file 22: — Chemosensitivity of A2780 and OVCAR8 ovarian cancer cells which are stably transfected with LacZ (control) or HOTAIR. Cells were treated with cisplatin (0.5–25 μM) or carboplatin (10–160 μM) for 3 days and analysed by the cell survival MTT assay (Sigma). (PDF 397 kb) [file 13073_2015_233_MOESM22_ESM.pdf]
