# Supplementary material for: HOTAIR and its surrogate DNA methylation signature indicate carboplatin resistance in ovarian cancer
Source: Genome Med. 2015 Oct 24;7:108. doi: 10.1186/s13073-015-0233-4 (PMC4619324; doi:10.1186/s13073-015-0233-4)
Supplement: Additional file 8: — Hazard ratios for relapse and death for patients who received carboplatin treatment (a) and patients who did not receive carboplatin treatment (b) in the original INNSBRUCK set. (PDF 183 kb) [file 13073_2015_233_MOESM8_ESM.pdf]

Additional data file 8. Hazard Ratios for Relapse and Death. (A) Patients with Carboplatin treatment **(B)** patients without Carboplatin treatment ("INNSBRUCK" set).

A

**Characteristics**	**Relapse** Hazard Ratio (95% CI)	**P Value***	**Death** Hazard Ratio (95% CI)	**P Value***
**Age**		0.745		0.055
> 62.7 yrs. Vs <62.7 yrs.	1.11 (0.59-2.11)		1.80 (0.99-3.28)	
**FIGO stage**		**0.002**		**0.002**
III/IV vs I/II	9.28 (2.21-38.86)		4.41 (1.73-11.26)	
**Tumor grade**		0.509		0.371
III vs. I/II	1.25 (0.64-2.43)		1.32 (0.72-2.42)	
**Residual disease after surgery**		**0.014**		**0.010**
disease vs. no disease	3.02 (1.25-7.31)		2.76 (1.28-5.98)	
**Histology**		0.120		0.108
non-serous vs. Serous	0.59 (0.31-1.15)		0.61 (0.34-1.11)	
***HOTAIR* RNA expression**		**< 0.001**		**< 0.001**
pos. vs neg.	4.46 (2.14-9.31)		4.02 (2.11-7.64)	
HOTAIR* multivariate*				
pos. vs neg.	3.38 (1.53-7.44)	**0.003**	3.64 (1.78-7.42)	**<0.001**

B

**Characteristics**	**Relapse** Hazard Ratio (95% CI)	**P Value***	**Death** Hazard Ratio (95% CI)	**P Value***
**Age**		0.375		0.012
> 62.7 yrs. Vs <62.7 yrs.	1.34 (0.70-2.55)		2.05 (1.17-3.60)	
**FIGO stage**		0.053		0.136
III/IV vs I/II	2.25 (0.99-5.09)		1.62 (0.86-3.07)	
**Tumor grade**		**0.002**		**0.027**
III vs. I/II	2.70 (1.43-5.11)		1.93 (1.08-3.47)	
**Residual disease after surgery**		**0.003**		**0.005**
disease vs. no disease	3.10 (1.45-6.62)		2.47 (1.31-4.67)	
**Histology**		0.063		0.712
non-serous vs. Serous	0.54 (0.29-1.03)		0.90 (0.51-1.58)	
***HOTAIR* RNA expression**		0.966		0.774
pos. vs neg.	0.99 (0.53-1.84)		0.92 (0.52-1.62)	
HOTAIR* multivariate*				
pos. vs neg.	1.13 (0.57-2.23)	0.722	0.97 (0.52-1.80)	0.932

* P values were calculated using Cox-Regression
** multivariate analysis included stage, grade, residual disease and *HOTAIR*
