# Supplementary material for: HOTAIR and its surrogate DNA methylation signature indicate carboplatin resistance in ovarian cancer
Source: Genome Med. 2015 Oct 24;7:108. doi: 10.1186/s13073-015-0233-4 (PMC4619324; doi:10.1186/s13073-015-0233-4)
Supplement: Additional file 9: — Hazard ratios (HR) for death of patients who received carboplatin-based treatment in the GRONINGEN set and had RNA available to analyze HOTAIR. (PDF 138 kb) [file 13073_2015_233_MOESM9_ESM.pdf]

Additional data file 9. Hazard Ratios (HR) for death of patients who received Carboplatin-based treatment in the "GRONINGEN" Set and had RNA available to analyze *HOTAIR* (n=157). Hazard ratio (with 95% confidence intervals), likelihood ratio test P-value, and number of data values, for various predictive factors in the Groningen set for a follow up period of 5 years (no patients were followed up for more than 5 years). *HOTAIR*-multivariate denotes multivariate analysis adjusted for stage and size of residual tumor.

	5 yrs		
Factor	HR (95%CI)	P	n
Age	1.01 (0.99-1.02)	0.389	170
Stage	2.08 (1.33-3.25)	**0.001**	170
Grade	1.15 (0.86-1.54)	0.333	151
Residual Tumor	1.79 (1.34-2.39)	**<0.001**	158
HOTAIR positivity	1.86 (1.21-2.88)	**0.004**	157
HOTAIR positivity multivariate	1.63 (1.04-2.56)	**0.032**	146
