# Supplementary material for: HOTAIR and its surrogate DNA methylation signature indicate carboplatin resistance in ovarian cancer
Source: Genome Med. 2015 Oct 24;7:108. doi: 10.1186/s13073-015-0233-4 (PMC4619324; doi:10.1186/s13073-015-0233-4)
Supplement: Additional file 15: — Performance of the DNAme signature in the primary INNSBRUCK carboplatin-treated ovarian cancer set and TCGA set. (PDF 215 kb) [file 13073_2015_233_MOESM15_ESM.pdf]

Additional data file 15A. DNAme signature in primary Carboplatin-treated "INNSBRUCK" ovarian cancer set (n=63). Cox-regression Hazard ratio (with 95% confidence intervals), likelihood ratio test P-value, and number of data values, for various predictive factors in the subset of 63 Carboplatin treated ovarian cancer patients, using the full follow-up period (maximum 17 years) and for the case of a 5 year observation window (all events censored at 5 years). *HOTAIR*-DNAme-multivariate denotes multivariate analysis adjusted for age, stage and size of residual tumor.

[†] *HOTAIR* DNAme correlation score was used (not the binarised score).

[$] *HOTAIR* DNAme correlation score was binarised into high and low groups according to a cut-off value as described in Supplementary Methods.

	Full period			<5 yrs		
Factor	HR (95%CI)	P	n	HR (95%CI)	P	n
Age	1.51 (1.11-2.05)	**0.008**	63	1.70 (1.18-2.47)	**0.003**	63
Stage	1.88 (1.28-2.77)	**0.001**	63	1.88 (1.20-2.96)	**0.005**	63
Grade	1.39 (0.79-2.46)	0.25	59	1.01 (0.53-1.93)	0.98	59
Residual Tumor	3.06 (1.90-4.93)	**<0.001**	60	3.18 (1.85-5.46)	**<0.001**	60
HOTAIR-DNAme[†]	1.56 (1.18-2.07)	**0.001**	63	1.76 (1.27-2.45)	**<0.001**	63
HOTAIR-DNAme[$]	2.34 (1.29-4.26)	**0.004**	63	3.07 (1.49-6.31)	**0.001**	63
HOTAIR-DNAme[†] multivariate	1.54 (1.09-2.17)	**0.01**	60	1.72 (1.14-2.59)	**0.009**	60
HOTAIR-DNAme[$] multivariate	2.35 (1.19-4.63)	**0.01**	60	2.76 (1.24-6.18)	**0.01**	60

Additional data file 15B. DNAme signature in the independent "TCGA" set (n=316; Carboplatin based treatment; excluding Cisplatin treated patients). Cox-regression Hazard ratio (with 95% confidence intervals), likelihood ratio test P-value, and number of data values, for various predictive factors in the TCGA set, using the full follow-up period (maximum 10.8 years) and for the case of maximum 5 years follow-up period (all observations after 5 years are censored at 5 years). *HOTAIR*-DNAme-multivariate denotes multivariate analysis adjusted for age, stage and size of residual tumor (full period) and for age, stage, grade and size of residual tumor for 5 year observation window. All Cox-regressions were stratified by the medical health center the patients came from to account for inter-center differences in survival distributions.

† *HOTAIR* DNAme correlation score was used (not the binarised score).

$ *HOTAIR* DNAme correlation score was binarised into high and low groups according to a cut-off value as described in Supplementary Methods.

	Full period			<5 yrs		
Factor	HR (95%CI)	P	n	HR (95%CI)	P	n
Age	1.20 (1.00-1.43)	0.05	275	1.27 (1.05-1.53)	**0.01**	275
Stage	1.70 (1.12-2.56)	**0.012**	275	1.54 (1.00-2.38)	**0.05**	275
Grade	1.23 (0.71-2.12)	0.46	268	1.65 (0.87-3.10)	**0.12**	268
Residual Tumor	1.39 (0.98-1.96)	**0.062**	246	1.29 (0.91-1.85)	**0.16**	246
HOTAIR-DNAme†	1.44 (1.19-1.73)	**<0.001**	275	1.55 (1.27-1.88)	**<0.00001**	275
HOTAIR-DNAme$	1.49 (1.07-2.10)	**0.02**	275	1.77 (1.22-2.58)	**0.003**	275
HOTAIR-DNAme† multivariate	1.32 (1.07-1.61)	**0.01**	246	1.45 (1.17-1.80)	**<0.001**	241
HOTAIR-DNAme$ multivariate	1.68 (1.10-2.56)	**0.02**	241	1.91 (1.23-2.98)	**0.004**	241
