# Supplementary material for: HOTAIR and its surrogate DNA methylation signature indicate carboplatin resistance in ovarian cancer
Source: Genome Med. 2015 Oct 24;7:108. doi: 10.1186/s13073-015-0233-4 (PMC4619324; doi:10.1186/s13073-015-0233-4)
Supplement: Additional file 18: — Performance of the DNAme signature in the carboplatin (a) and cisplatin (b) treated EUROPE set. (PDF 212 kb) [file 13073_2015_233_MOESM18_ESM.pdf]

Additional data file 18A. DNAme signature in the Carboplatin treated "EUROPE" set (n=121). Cox-regression Hazard ratio (with 95% confidence intervals), likelihood ratio test P-value, and number of data values, for various predictive factors in the EUROPE set for full period and 5 years follow-up period (all observations after 5 years are censored at 5 years). *HOTAIR*-DNAme-multivariate denotes multivariate analysis adjusted for stage and size of residual tumor.

† *HOTAIR* DNAme correlation score was used (not the binarised score).

$ *HOTAIR* DNAme correlation score was binarised into high and low groups according to a cut-off value as described in Supplementary Methods.

	Full period			<5 yrs		
Factor	HR (95%CI)	P	n	HR (95%CI)	P	n
Age	1.10 (0.90-1.33)	0.35	121	1.08 (0.86-1.34)	0.51	121
Stage	1.50 (1.19-1.89)	**<0.001**	121	1.61 (1.24-2.10)	**<0.001**	121
Grade	1.22 (0.96-1.55)	0.097	121	1.25 (0.96-1.64)	0.095	121
Residual Tumor	1.28 (1.04-1.57)	**0.016**	121	1.21 (0.96-1.52)	0.097	121
HOTAIR-DNAme†	1.16 (0.94-1.43)	0.15	121	1.13 (0.90-1.42)	0.31	121
HOTAIR-DNAme$	1.74 (1.11-2.73)	**0.01**	121	1.98 (1.20-3.26)	**0.008**	121
HOTAIR-DNAme† multivariate	1.16 (0.94-1.44)	0.17	121	1.19 (0.94-1.51)	0.14	121
HOTAIR-DNAme$ multivariate	1.63 (1.04-2.56)	**0.03**	121	1.86 (1.12-3.07)	**0.01**	121

Additional data file 18B. DNAme signature in the Cisplatin treated "EUROPE" set (n=85). Cox regression Hazard ratio (with 95% confidence intervals), likelihood ratio test P-value, and number of data values, for various predictive factors in the EUROPE set for full period and 5 years follow-up period (all observations after 5 years are censored at 5 years). *HOTAIR*-DNAme-multivariate denotes multivariate analysis adjusted for stage and size of residual tumor. We note that the *HOTAIR* DNAme correlation score was used, not the binarised score.

† *HOTAIR* DNAme correlation score was used (not the binarised score).

$ *HOTAIR* DNAme correlation score was binarised into high and low groups according to a cut-off value as described in Supplementary Methods.

	Full period			<5 yrs		
Factor	HR (95%CI)	P	n	HR (95%CI)	P	n
Age	1.34 (1.03-1.74)	0.29	85	1.30 (0.98-1.72)	0.063	85
Stage	2.29 (1.60-3.27)	**<0.001**	85	2.18 (1.51-3.14)	**<0.001**	85
Grade	1.05 (0.82-1.34)	0.72	85	0.99 (0.76-1.30)	0.97	85
Residual Tumor	1.75 (1.32-2.30)	**<0.001**	85	1.76 (1.31-2.36)	**<0.001**	85
HOTAIR-DNAme†	0.85 (0.65-1.10)	0.22	85	0.93 (0.70-1.23)	0.62	85
HOTAIR-DNAme$	0.80 (0.48-1.35)	0.41	85	0.91 (0.52-1.58)	0.73	85
HOTAIR-DNAme† multivariate	0.90 (0.69-1.18)	0.17	85	0.98 (0.73-1.30)	0.88	85
HOTAIR-DNAme$ multivariate	0.80 (0.47-1.35)	0.40	84	0.90 (0.51-1.58)	0.72	85
