# Supplementary material for: HOTAIR and its surrogate DNA methylation signature indicate carboplatin resistance in ovarian cancer
Source: Genome Med. 2015 Oct 24;7:108. doi: 10.1186/s13073-015-0233-4 (PMC4619324; doi:10.1186/s13073-015-0233-4)
Supplement: Additional file 19: — Performance of the DNAme signature in the carboplatin-treated set from the ROCHESTER-MAYO set. (PDF 201 kb) [file 13073_2015_233_MOESM19_ESM.pdf]

Additional data file 19. DNAme signature in the Carboplatin treated set from "ROCHESTER-MAYO" (n=174). Cox regression Hazard ratio (with 95% confidence intervals), likelihood ratio test P-value, and number of data values, for various predictive factors in the MAYO set for full period and 5 years follow-up period (all observations after 5 years are censored at 5 years). *HOTAIR*-DNAme-multivariate denotes multivariate analysis adjusted for stage and size of residual tumor.

[†] *HOTAIR* DNAme correlation score was used (not the binarised score).

[$] *HOTAIR* DNAme correlation score was binarised into high and low groups according to a cut-off value as described in Supplementary Methods.

	Full period			<5 yrs		
Factor	HR (95%CI)	P	n	HR (95%CI)	P	n
Age	1.21 (1.00-1.46)	0.05	174	1.21 (0.98-1.49)	0.08	174
Stage	1.53 (1.08-2.15)	**0.02**	174	1.62 (1.11-2.38)	**0.01**	174
Grade	1.04 (0.76-1.41)	0.81	169	1.17 (0.82-1.66)	0.39	169
Residual Tumor	1.34 (1.12-1.60)	**0.001**	136	1.33 (1.09-1.63)	**0.004**	136
HOTAIR-DNAme[†]	1.09 (0.91-1.32)	0.22	174	1.18 (0.95-1.45)	0.13	174
HOTAIR-DNAme[$]	1.03 (0.70-1.52)	0.87	174	1.19 (0.77-1.85)	0.44	174
HOTAIR-DNAme[†] multivariate	1.31 (1.03-1.66)	**0.03**	136	1.45 (1.12-1.88)	**0.004**	136
HOTAIR-DNAme[$] multivariate	1.31 (0.83-2.07)	0.23	136	1.57 (0.95-2.60)	**0.076**	136
